# MSF experiences of providing multidisciplinary primary level NCD care for Syrian refugees and the host population in Jordan: an implementation study guided by the RE-AIM framework

**DOI:** 10.1186/s12913-021-06333-3

**Published:** 2021-04-26

**Authors:** Éimhín Ansbro, Tobias Homan, Jamil Qasem, Karla Bil, Mohammed Rasoul Tarawneh, Bayard Roberts, Pablo Perel, Kiran Jobanputra

**Affiliations:** 1 Centre for Global Chronic Conditions, London School of Hygiene and Tropical Medicine, London, UK; 2 Médecins sans Frontières, Amman, Jordan; 3 Médecins sans Frontières, Irbid, Jordan; 4 Médecins sans Frontières, Amsterdam, The Netherlands; 5 Noncommunicable Disease Directorate, Ministry of Health, Amman, Jordan; 6 Médecins sans Frontières, London, UK

**Keywords:** Non communicable disease, Diabetes, Hypertension, Cardiovascular disease, Humanitarian, Conflict, Effectiveness, Refugee, Syria, Jordan, Programme, RE-AIM, Evaluation, Implementation

## Abstract

**Background:**

In response to the rising global NCD burden, humanitarian actors have rapidly scaled-up NCD services in crisis-affected low-and-middle income countries. Using the RE-AIM implementation framework, we evaluated a multidisciplinary, primary level model of NCD care for Syrian refugees and vulnerable Jordanians delivered by MSF in Irbid, Jordan. We examined the programme’s *Reach*, *Effectiveness*, *Adoption* and acceptance, *Implementation* and *Maintenance* over time.

**Methods:**

This mixed methods retrospective evaluation, undertaken in 2017, comprised secondary analysis of pre-existing cross-sectional household survey data; analysis of routine cohort data from 2014 to 2017; descriptive costing analysis of total annual, per-patient and per-consultation costs for 2015–2017 from the provider-perspective; a clinical audit; a medication adherence survey; and qualitative research involving thematic analysis of individual interviews and focus group discussions.

**Results:**

The programme enrolled 23% of Syrian adult refugees with NCDs in Irbid governorate. The cohort mean age was 54.7 years; 71% had multi-morbidity and 9.9% self-reported a disability. The programme was acceptable to patients, staff and stakeholders. Blood pressure and glycaemic control improved as the programme matured and by 6.6 mmHg and 1.12 mmol/l respectively within 6 months of patient enrolment. Per patient per year cost increased 23% from INT$ 1424 (2015) to 1751 (2016), and by 9% to 1904 (2017). Cost per consultation increased from INT$ 209 to 253 (2015–2017). Staff reported that clinical guidelines were usable and patients’ self-reported medication adherence was high. Individual, programmatic and organisational challenges to programme implementation and maintenance included the impact of war and the refugee experience on Syrian refugees’ ability to engage; inadequate low-cost referral options; and challenges for MSF to rapidly adapt to operating in a highly regulated and complex health system. Essential programme adaptations included refinement of health education, development of mental health and psychosocial services and addition of essential referral pathways, home visit, physiotherapy and social worker services.

**Conclusion:**

RE-AIM proved a valuable tool in evaluating a complex intervention in a protracted humanitarian crisis setting. This multidisciplinary programme was largely acceptable, achieving good clinical outcomes, but for a limited number of patients and at relatively high cost. We propose that model simplification, adapted procurement practices and use of technology could improve cost effectiveness without reducing acceptability, and may facilitate replication.

**Supplementary Information:**

The online version contains supplementary material available at 10.1186/s12913-021-06333-3.

## Background

In recent years, humanitarian actors have had to rapidly scale-up NCD services in response to the rising global burden of NCDs and to the specific crises involving middle-income countries with high NCD burdens [1, 2]. There is strong evidence on cost-effective, primary care-based clinical management of NCDs in stable, high-income countries. However, there are limited clinical and programmatic tools available to guide NCD interventions in low- and middle-income countries (LMICs) and even less addressing those affected by humanitarian crises and forced displacement [3–5]. The literature describing NCD programme implementation or evaluation in humanitarian settings is especially limited [6, 7]. In response to this gap, humanitarian actors, including the medical humanitarian non-governmental organisation (NGO) Médecins sans Frontières, have adapted their traditional approaches to care for chronic disease, developing clinical and programmatic guidance, monitoring and evaluation tools and an NCD emergency response kit [8, 9]. As they have gained increasing experience of NCD care delivery, some humanitarians have called for the sustainability of NCD interventions to be considered in their design and for handover to local health structures to occur during protracted crises [2].

The challenges of evaluating interventions in humanitarian settings are well known [10–13]. Traditional experimental methods may be unfeasible or even unethical to implement in such settings; target populations are vulnerable and humanitarian contexts are dynamic and potentially insecure; and there may be limited skills, time and funding available for research and evaluation within humanitarian organisations [11]. There is a clear need to develop robust strategies to evaluate programmes in disaster settings that are rapid, pragmatic and that impose minimal burden on implementing teams [13]. RE-AIM is an implementation research framework that has been used successfully for planning and evaluating interventions in both high-income and LMIC settings [14]. To the best of our knowledge, it has not yet been comprehensively applied to a humanitarian intervention. It was designed to facilitate the translation of research into practice and to improve the reporting of key elements essential for successful programme implementation, at both individual- and organisational-levels [14–18]. Using mixed methods, the framework assesses programmes under five key domains: *reach, effectiveness, adoption, implementation,* and *maintenance* (Table 1).

**Table 1:** Example indicators and data method/source based on the RE-AIM domains

Objective / domain (questions)	Sub-domain	Indicator	Methods (a methodology may feature under several headings)
**Reach** • Target population reached?	**Coverage**	▪ % people among the target population eligible for programme and number served by the programme ▪ Prevalence of NCD and MH comorbidity^b^	• Existing MSF household survey ^a^ • Routine cohort data • Qualitative data
**“Effectiveness”/ Quality of Care** ▪ Trends in clinical outcomes and quality indicators? ▪ Perceived benefits/unintended consequences from a patient and provider perspective?	**Clinical Outcomes**	▪ % HTN patients with most recent BP < 140/90 mmHg, 6 & 12 months post enrolment and trend from baseline^b^ ▪ % Patients with diabetes with last HbA1c < 8.0% 6 & 12 months post enrolment and trend from baseline^b^ ▪ % Patients who report decreased/quitting smoking	• Routine cohort data • Qualitative data
	**Quality Indicators**	▪ % active^c^ CVD patients prescribed a statin ▪ % COPD/ asthma patients with inhaler technique check documented ▪ Trend in defaulters^c^ and deaths as a proportion of active cohort	• Clinical audit • Routine cohort data
	**Perceived Effectiveness**	▪ Patients’ and providers’ perspectives on effectiveness of programme components (clinical review, medications, HE, HLO, MHPSS, HV)	• Qualitative data
**Adoption/ acceptance** ▪ Care model accessible and acceptable to patients, providers, organisation and community? ▪ Guideline acceptable to staff?	**Accessibility/ acceptability**	▪ Availability and accessibility / barriers to access ▪ Acceptability/usability of NCD guideline ▪ Self-reported medication adherence and medication beliefs	▪ Routine cohort data ▪ Qualitative data ▪ Self-report medication adherence questionnaire
	**Adoption/participation**	▪ Description of intervention location, cadres of staff and qualifications ▪ Experience of receiving and providing NCD care, use of clinical guideline ▪ How participation influenced patient/staff well-being and/or work practices	▪ Routine cohort data ▪ Qualitative data
**Implementation** ▪ Intervention delivered as intended? ▪ Facilitators and barriers to implementing the programme? ▪ Essential components and adaptations necessary? ▪ Implementation costs?	**Fidelity of programme delivery**	▪ % DM patients with micro-albuminuria or urinary protein tested ▪ % Active^c^ cohort attending a health education session at last clinical visit ▪ No. of MHPSS group sessions monthly during reporting period	▪ Clinical audit ▪ Routine cohort data
	**Adaptations**	• NCD care adaptations to local setting (e.g. cultural; dietary, exercise) • Programme adaptations related to humanitarian setting e.g. response to patients’ psychosocial needs	▪ Qualitative data
	**Cost**	• Staff time; • Capital and recurrent implementation costs^b^	▪ Qualitative data ▪ Medicine/supply/ staff costs^b^ ▪ Staff time estimates
**Maintenance** • Challenges and facilitators for patients to stay in programme? • Organisational challenges, and costs; adaptations made to maintain programme?	**Individual Level**	• % Patients active^c^ 6 months post enrolment^b^ • Self-reported medication adherence rates • Key challenges in altering lifestyle (diet, exercise, smoking)	▪ Routine cohort data ▪ Clinical Audit ▪ Qualitative data ▪ Medicine/supply/staff costs^b^ ▪ Staff time estimates ▪ Self-report medication adherence questionnaire
	**Organisational Level**	• Measures of cost of maintenance^b^ • Institutionalisation of the programme/modifications made for maintenance • Alignment with organisational mission	

Key: *BP* blood pressure, *COPD* chronic obstructive pulmonary disease, *CVD* cardiovascular disease, *HbA1c* glycosylated haemoglobin, *HLO* humanitarian liaison officer, *HV* home visit, *MH* mental health, *MHPSS* mental health and psychosocial support, *NCD* non-communicable disease

^a^Relevant methods and results are reported in Rehr et al. [19]

^b^Detailed methods and results are reported in linked papers [20, 21]

^c“^Active patients” means continued to attend the service and not exited [i.e. died, departed the area or defaulted (i.e. have not attended for more than 90 days since their last planned appointment)]

The Syrian conflict, now in its tenth year, continues to devastate the Syrian people. Since 2011, over 6.1 million Syrians have been internally displaced, while over 6.6 million have fled as refugees, mostly into surrounding countries [22]. Jordan currently hosts almost 670,000 Syrian refugees registered with the United Nations High Commissioner for Refugees (UNHCR). Globally, it ranks second only to Lebanon in the number of refugees it hosts relative to the national population [22, 23].

NCDs have been responsible for more deaths than communicable diseases in Syria for several decades, causing 77% of mortality before the conflict [24, 25] Therefore, host country and humanitarian actors have had to tackle the high NCD burden amongst Syrian refugees [23, 26–28]. In Jordan, the Ministry of Health (MOH) has been strengthening NCD care at primary level to address the rising NCD burden among its own population. At the time of this study, NCDs were diagnosed and monitored by family medicine specialists at MOH comprehensive primary centres while medication refills were provided by non-specialist doctors at primary health centre level. UNHCR funded registered Syrian refugees to access MOH primary care services and limited referral services. However, financial barriers (including the addition of user co-payments from 2014, which have varied over time reaching full “foreigner” rate by 2018), complex care pathways and referral systems, and limited health facility capacity have impeded refugees’ access to these services [29]. The burden, access issues and the broader health system response to Syrian refugees’ NCD needs in Jordan are well documented [19, 29–31]. However, little is known about the content or quality of current NCD programming, either within the MOH or parallel humanitarian health systems.

Since 2014, Médecins sans Frontières (MSF), a humanitarian medical organisation, has supported the Jordanian health system by providing multidisciplinary, primary level NCD care to Syrian refugees and the vulnerable host population in Irbid, north Jordan. In response to the urgent need for evidence to guide humanitarian actors in tackling NCDs in complex settings, we undertook a mixed methods evaluation of the MSF programme. We hoped to learn lessons to both improve the current care model and to inform the design of future NCD programmes in Jordan and elsewhere. Detailed analyses of cohort, qualitative and costing data are reported in separate papers [20, 21, 32]. The aim of this paper was to summarise the full evaluation, which used the RE-AIM implementation framework to examine the *Reach*; *Effectiveness*; *Adoption* and acceptance of the programme; *Implementation* fidelity, adaptations and costs*;* and programme *Maintenance* over time [33].

## Methods

This retrospective mixed methods evaluation of the MSF NCD programme in Irbid comprised secondary analysis of data from a pre-existing cross-sectional household survey [19], analysis of routine cohort data, a descriptive costing study, a clinical audit, a self-administered medication adherence survey and qualitative research. It was undertaken in late 2017 and covered the study period December 2014 to December 2017. This paper draws together the findings from all methodologies under the RE-AIM framework. Example indicators, based on the RE-AIM domains, and the relevant methods and data used to determine them are presented in Table 1. The full list is available in Supplementary Material 1.

### Study setting

The study was conducted in Irbid, the second largest city in Jordan. Irbid governorate hosted over 165,000 Syrian refugees who were mostly urban-based [34]. MSF commenced an NCD service within a Ministry of Health (MOH) primary care facility in Irbid in December 2014 serving non-camp dwelling Syrian refugees and the vulnerable Jordanian host community. A second site in the city was opened within a local NGO clinic in April 2016. The MSF service was vertical, operating in parallel to the pre-existing activities at each site rather than integrating with them. Medicines, consultations and laboratory investigations were provided free-of-charge to patients. The cohort size was capped by MSF at approximately 4000 for operational and cost reasons and the two sites were later amalgamated in 2019.

### Intervention

Detailed descriptions of the context, the intervention and a programme timeline are appended in the supplementary material (S2 and S3). In brief, this was a multi-disciplinary, primary care model, which used context-adapted clinical guidelines, generic medications in line with the World Health Organization (WHO) Essential Medicines list and task sharing.

#### Enrolment

Eligibility for enrolment required both medical and social indications. The target medical conditions were: hypertension (HTN), established cardiovascular disease (CVD) [angina, myocardial infarction, ischaemic stroke, transient ischaemic attack, peripheral vascular disease, congestive heart failure], diabetes mellitus (DM) type I or II, chronic obstructive pulmonary disease (COPD), asthma or hypothyroidism). Hereafter, these are referred to as “target NCDs”. Social indications included being a Syrian refugee (either registered or unregistered with UNHCR), a refugee of other origin or a vulnerable member of the Jordanian host population. Jordanians were considered vulnerable if they either lacked Jordanian national health insurance (and were therefore subject to co-payments to access MOH care) or were of low socioeconomic status. This was assessed using “vulnerability criteria” developed by the programme. Enrolment criteria changed over time, for example isolated hypothyroidism was removed and vulnerability criteria were adapted for ease of implementation. Enrolment was not limited by place of residence or age. Most patients presented with established, self-reported diagnoses; new diagnoses were made based on the MSF NCD guideline [8].

#### Service description

The multidisciplinary team initially included non-specialist doctors, nurses, health educators, pharmacy and reception staff, who provided appointment-based medical consultation, health education and behaviour change counselling, supported by a local management team and a coordination team in Amman. The service evolved to also incorporate individual- and group-based mental health and psychosocial support (MHPSS), social work, physiotherapy and a home visit service for house-bound patients, with the addition of counsellors, a humanitarian liaison officer, a home visit doctor and nurses, a physiotherapist and specialist family medicine practitioners. Facility-based services were provided 6 days per week from 8 am to 2 pm, while the home visit service operated on 6 days within a ten-mile radius of the clinics. By 2017, the team had introduced task sharing of some review visits. Further detail is available below and in Supplementary Material (S2 and S3).

### Study design

The RE-AIM domains were defined with reference to the relevant literature [14–17] and with some adaptations specific to this evaluation. *Reach* was defined as coverage of the NCD service and its components to the intended target population, with a focus on MHPSS services. RE-AIM defines *effectiveness* as the impact of an intervention on important outcomes, including potential negative effects, quality of life and costs. *Effectiveness w*as determined by examining: 1) trends in intermediate clinical outcomes, 2) quality of care indicators, 3) perceived benefits, unintended consequences and behavioural outcomes, and 4) economic outcomes. *Adoption / acceptance* were explored in relation to the organisation, setting, staff and patients and included changes to behaviour and practice. The *Adoption* domain is usually a “setting-level” outcome, defined in the literature in terms of absolute number, proportion, and representativeness of settings and intervention agents who are willing to initiate a program. Since this definition was not relevant to the MSF programme, as there was no choice for staff or settings to take part, we adapted this domain to cover patient adoption of the programme, including access and acceptability. *Implementation* of the NCD service was explored in relation to each programme component. We examined the fidelity of guideline implementation and its usability; the adaptation of structures, processes and tools; and the costs of implementation. *Maintenance* referred to the continued implementation of the NCD service over time by patients, the programme team and the organisation. The specific indicators and methodologies used to operationalize these definitions are listed in Table 1 and Supplementary Material 1. Qualitative and quantitative data from the various data sources were synthetized using the RE-AIM framework.

### Study participants, data collection and analysis

#### Household survey

To explore programme coverage, we used previously reported data from a Household Access and Utilisation Survey conducted by MSF in Irbid governorate, north Jordan in 2016. MSF undertook the survey to inform health service planning for the refugee population. They estimated the prevalence of NCDs and NCD multi-morbidity and determined factors associated with high NCD prevalence. Data collection and analysis, using a two-stage cluster design, are described in detail elsewhere [19].

#### Retrospective cohort study

To explore cohort demographics, NCD prevalence and service use, we analysed data from all patients who ever attended an enrolment visit in MSF’s NCD clinics from December 2014 to December 2017. Descriptive statistics were used to examine patient demographics and process indicators. We explored trends in intermediate clinical outcomes and treatment interruption from programme and patient perspectives, and the factors associated with these trends. We included patients 18 years and older with hypertension and/or diabetes type II (DM II), exploring control of systolic blood pressure (SBP < 140 mmHg) and glycaemia [fasting capillary blood glucose (FBG) ≤ 180 mg/dL or HbA1c < 8%] [21]. We plotted monthly means for each outcome (SBP, FBG, HbA1c or treatment delay) and the proportion of monthly visits at which targets were achieved. We used Generalised Linear Mixed-Effects Models (GLMM) to explore factors associated with each outcome. The analysis is elaborated on in our related paper [21]. Routine paper-based clinical data were collected by MSF data clerks and entered into a bespoke password-protected Microsoft Excel software database. Cohort data from both clinical sites were aggregated and analysed using R v1.0.136 (R, Boston, MA 02210, USA).

#### Costing study

A descriptive costing analysis from the provider perspective aimed to explore the annual total, per patient and per consultation costs for the Irbid NCD programme for 2015, 2016 and 2017. The analysis delineated capital and recurrent costs incurred at clinic- and project team-levels in Irbid and coordination team-level in Amman. Recurrent costs included human resources, medicines and equipment, building and vehicle costs, and training and supervision. We excluded direct or indirect patient-incurred costs. The analysis is described in detail in our companion paper [20].

#### Clinical audit

The clinical audit aimed to explore programme quality by examining fidelity of guideline implementation. We used a random selection of paper files from patients enrolled at least 12 months in the programme. Data were collected in August 2017 by programme medical staff on a paper-based checklist and entered into a purpose-designed Excel spread-sheet. We used process indicators analysed using descriptive statistics (Table 1; S1).

#### Medication adherence survey

A convenience sample of 300 consenting patients aged 18 or over attending either MSF clinic site during a 2-week period in September 2017 was selected (Supplementary material S4). The 17-item adherence survey included demographic information and pre-existing self-reported medication adherence and beliefs measures: the Medication Adherence Report Scale-5 item (MARS-5) and the Beliefs About Medicines Questionnaire (BMQ). Two trained data collectors took written informed consent from patients, who self-filled the survey in Arabic. Data collectors assisted those with limited literacy. Paper data were held securely and were entered into a purpose-designed Excel tool. Analysis included descriptive statistics and multivariate logistic regression.

#### Qualitative study

The methods are described in detail in Supplementary material S5. In brief, this involved two same-sex focus group discussions (FGDs) with eight Syrian adult patients each and 40 individual semi-structured interviews, including 16 with adult Syrian and Jordanian patients, 18 with MSF staff, and seven with key stakeholders, including staff from the MOH and other international NGOs involved in NCD care delivery. Data were collected by two local researchers and the principal researcher, in Arabic and English, in August 2017. EA and a second analyst (LM) performed thematic analysis, based on the RE-AIM framework, using a combination of inductive and deductive coding.

The findings are reported in accordance with the Consolidated Criteria for Reporting Qualitative Research checklist for transparency [35]. Mental health and social suffering emerged as prominent, data-derived themes and have been reported in detail separately [32]. The remaining themes are reported here.

This study protocol was granted approval by the MSF Ethics Review Board and LSHTM Ethics Committee. Written authorisation to implement the study was obtained from the Ministry of Health of Jordan.

## Results

The results are presented according to each RE-AIM domain and subdomain (Table 1). These have been somewhat reordered compared to our protocol to facilitate logical presentation.

### Reach

We explored the numbers eligible for the programme, numbers reached and representativeness of those reached. The project proposal defined the target population as Syrians with target NCDs resident in Irbid governorate. To explore access and coverage, MSF performed a Household Access and Utilisation Survey in 2016. Results showed one fifth of surveyed adult Syrians in Irbid governorate self-reported at least one NCD targeted by MSF (21.8% of 8041 surveyed adults aged 18 or over). UNHCR and others estimated that 95% of refugees resident in Irbid governorate in 2017 were registered with UNHCR (*n* = 135,144 in December 2017) of whom 48.7% were adults aged ≥18 years [19, 36]. This implies there were 142,256 total refugees with 69,278 ≥ 18 years. Applying the household survey figure of 21.8% meant 15,102 Syrian refugees ≥18 years in Irbid governorate had an MSF-targeted NCD and were therefore eligible for enrolment in the programme. Since 3531 Syrian adult patients were ever-enrolled (limited by the cap on cohort size), 23.4% of the target population was reached by this MSF programme [37]. Syrians resident in other governorates were also eligible. Patients were enrolled on a first-come-first-served basis and news of the programme quickly spread by word of mouth. The Jordanian government required that international medical providers enrolled a varying proportion of the host community in their programmes. MSF defined its own ‘vulnerability’ criteria which took into account economic as well as social factors, with reference to the Jordanian Ministry of Social Welfare. The definition of eligibility (vulnerability) changed over time.

Retrospective data were analysed from 5045 patients ever enrolled during the study period. The cohort comprised 3664 (72.6%) Syrians, 1365 (27.1%) Jordanians and 16 (0.3%) refugees of other origins (Palestinian or Iraqi), who were middle-aged [mean 54.7 years (SD 15.7)] with multi-morbidity and relatively high rates of self-reported disability (9.9%). The majority (59.8%) were women and 71% (*n* = 3582) had two or more target NCD conditions, with hypertension (60.4%), type 2 diabetes (53.1%), cardiovascular disease (25.9%), hypothyroidism (7.6%) and asthma (7.0%) the most commonly treated conditions (Supplementary material S6). These findings are consistent with the MSF Household Access Survey, which reported a similar prevalence of target NCDs [19]. However, the MSF clinic cohort had greater rates of NCD multi-morbidity compared to the adults with NCDs in the household survey (71% vs. 44.7%). NCD risk factor levels were high at enrolment with obesity levels of 62.6%, self-reported smoking rates of 22.7%, and low or zero self-reported regular physical activity in 37.2% (Supplementary material S6). The reach of the MHPSS service is described below.

### Access, acceptance and adoption

Under this domain, we described the programme’s components, structures and staffing and we explored patient, provider and stakeholder perspectives on programme accessibility.

#### Accessibility

We considered access in terms of availability, cost and physical accessibility. MSF services were available to 23.4% of their targeted Syrian population. MSF took a “cohort approach” to their service provision and both MSF and Jordanian policy required services to also be delivered to the host population. MSF’s policy of providing free-of-charge care facilitated access for the enrolled Syrians and vulnerable Jordanians to medical consultation, consistent medication supply and laboratory testing. Patients only incurred transport and indirect costs, such as loss of income. Syrian interviewees, in particular, reported carefully balancing stretched household finances, and prioritising expenditure on transport costs for aspects of the MSF service they valued, such as medical consultations, over those for MHPSS, health education or laboratory visits. Some chose to purchase their preferred medications from other sources if not provided by MSF.

Patients reported the MSF clinics were also accessible in terms of distance, transport and convenience.

We also explored Syrian community members’ access to alternative, affordable primary level NCD services in north Jordan, since MSF’s future programme plans hinged on whether such a source of affordable NCD care was available.

*“…access to good quality care… that is reliable and regular and predictable…. I think that is a big challenge. Affordability is another challenge…”* MSF management staff member*.*

The MSF Household Access Survey corroborates our qualitative finding that cost was the main barrier to obtaining NCD care from other providers. Around a quarter of surveyed adult refugees with self-reported NCDs did not seek care when they felt it was needed. Only 10% reported poor availability as the reason, while the majority (60%) cited cost. Among those who received care, around half made a co-payment [19]. Interviewed MSF patients described their difficulty in obtaining a regular supply of affordable NCD medications before enrolling with MSF:

*“It’s difficult to buy the medicine always because I can’t afford it. Thank god when I registered at (the MSF clinic) … I started to have it free. Before I used to take from other places by small amounts of money (or) from the community pharmacy I paid it all.”* Syrian FGD participant*.*

Other international NGOs also provided NCD care to registered and unregistered refugees in Irbid governorate with some requiring co-payments. Registered refugees’ access to MOH primary care clinics was initially free-of-charge but increasing co-payments were introduced from 2014 and most interviewed patients described such co-payments, coupled with travel costs as unaffordable.

Despite the other available options, MSF staff reported they had a long waiting list of people wishing to access the MSF service. When asked how NCD patients in their community who were not enrolled in the MSF programme coped, interviewees reported that they skipped medications, shared with family or neighbours or purchased from private pharmacies:

Syrian patient: *“If there is a family that can’t bring medicine, we collect pills from here and here, so people help each other ... because there is extra. So people give to each other. I know a kid who takes insulin…I give to people. I’m forced to help people.”*

Staff perceived that most Jordanian patients did not, in fact, meet vulnerability inclusion criteria and could, therefore, access alternative free-of-cost services via national or military insurance. This was the case for all interviewed Jordanian patients.

We focused particularly on the theme of access to specialist referral services. In the middle-income setting of Jordan, secondary and tertiary care services were widely available within the public and private sector, including essential NCD referral services such as ophthalmology, endocrinology, cardiology, nephrology and emergency services. However, as described by our interviewees, accessing specialist services for NCD complications or other conditions via the humanitarian system referral pathway was complex, inconsistent and burdensome for patients, while accessing them directly was costly. In addition to funding primary level MOH access, UNHCR funded registered and unregistered refugees’ access to limited public and private specialist services via their implementing partner Jordan Health Aid Society (JHAS). JHAS played a gatekeeper role and interviewees from MSF and other NGOs perceived their decision-making process as “unhelpful” and lacking clear criteria:

*“We don’t really have any … clear structure dealing with (specialised secondary referrals). The identified system through JHAS and UNHCR, as the funding partner, is complex and lacks clarity and doesn’t always suit our patients.”* MSF clinical staff member.

MSF clinical staff could also refer patients to services provided by other NGOs but felt frustrated and disempowered by the lack of clarity and consistency regarding referral pathways, the lack of information returned by most referral services and lack of direct referral pathways to MOH specialist care. To address this, MSF had brokered agreements with other NGOs to provide retinopathy screening and angiography free-of-cost to patients as part of a defined short-term project. MSF, MOH and other interviewed stakeholders, suggested that encouraging other international NGOs to fund and implement similar services was the only way to fill the referral gap, since international funding was limited and dwindling.

#### Acceptance and adoption/participation

Under this domain, we described the programme location, cadres of staff and qualifications. During interviews we explored patients’, staff and stakeholders’ acceptance of the programme. With patients, we explored their sources of information and support; their experiences of receiving NCD care and how programme participation influenced their well-being.

Most programme elements were acceptable to patients, staff and stakeholders. Interviewed patients felt they received trusted, good quality care in a caring and respectful environment. Patients reportedly valued free-of-charge medications, regular laboratory and vital sign testing most highly but also valued healthy living advice and “encouragement” given by staff. One female patient reported:

“*(MSF is) honestly caring about the patient, caring about his appointments even the medication availability. We have never come here and told us that the medication is not available. Their performance is great.*”

Patients favourably compared their experience in the MSF clinic with their prior experiences at other NGO- or MOH-provided services. However, several expressed frustration at MSF narrow range of services and the limited provision of specialist care.

MSF national and international staff generally prided in their work for MSF:

*“…Syrians, we save their lives, … for me this service is like life… this disease is very difficult and chronic …and treatment costs a lot.”* Clinic staff member.

Clinical staff were mainly Jordanian medical and paramedical university graduates, many with previous NGO experience. They were committed to the MSF team and their patients and derived satisfaction from observing patients’ improvements.

*“I learned here how to see others’ problems… the disaster they are coming from…how we work here like a team or a family for the benefit of the patients; how you can give to the people…without taking, with nothing in return.”* Clinical staff member*.*

There was low turnover among clinical cadres other than non-specialist doctors, who tended to resign after gaining several months’ experience with MSF to pursue specialist training. This turnover was considered problematic by clinical supervisors, other staff and patients, all of whom valued continuity of care. A minority of staff expressed dissatisfaction with the perceived lack of promotion opportunities or job security (given the limited duration of MSF programmes), high workload and six-day working week. Interviewed stakeholders valued the programme since it relieved a significant burden on the MOH. Several called for it to be expanded in terms of coverage and scope (for example, by financing specialist referral care).

### Effectiveness

To evaluate *Effectiveness*, we examined clinical and quality indicators (Table 2) using retrospective analysis of routine clinical and programmatic data and clinical audit. Perceived effectiveness was explored using qualitative data.

**Table 2:** Effectiveness indicator results

	Result or comment
**a. Clinical Outcome Indicators**	
% ≥ 0.5 mmol/L reduction in total cholesterol from enrolment to last visit (those enrolled > = 90 days)	Among those with a cholesterol test who were in the cohort for at least 90 days (2585), 651 had ≥ reduction of 0.5 mmol/L in total cholesterol = 25.1%
% patients with asthma free from exacerbations/ admissions in previous 6 months	Among 382 patients with asthma, only 25 recorded exacerbations in total during the 3-year study period.
% patients who report decreased/quitting smoking	Not available as self-reported smoking category (stopped, decreased, increased, resumed, unchanged) was reported relative to the last appointment.
% patients who report increased levels of exercise from baseline	At each visit the category (active, inactive, moderately active, and moderately inactive) for recent activity behaviour was recorded. 3347 patients enrolled in the project at least 90 days had a first and last measurement. 610 (18.2%) had improved activity. 593 (17.7%) had worse activity. 2144 (64.1%) stayed the same. There was no significant improvement (chi sq. =0.284, *p* = 0.594).
Trend in referrals to another facility for acute complications/specialist care (% of active cohort)	Trend in referral by type of referral service and volume of referrals were analysed
**b. Quality (Process) Indicators**	
% recommended referrals to other services that are appropriate as per guideline	Not tested
% of active patients with CVD^a^ prescribed a statin	*N* = 369 (25.8%)
% of patients with CVD^a^ prescribed aspirin	*N* = 717 (50.1%)
% of patients with CVD^a^ prescribed at least one anti-hypertensive^b^ drug	*N* = 1007 (70.4%)
% of patients with asthma^c^ with inhaler technique check documented	*N* = 48 (94%)
No./% of times when appropriate clinical action taken based on clinical or laboratory findings	Among 130 randomly audited diabetic patient files, 100% had cholesterol checked; 73.8% (*n* = 82) had a CVD risk score subsequently calculated. Of these, 65.9% had a statin correctly prescribed (or not prescribed) according to MSF guidelines^d^.
Description of cohort deaths	2.6% (*n* = 139) of enrolled patients died by end of study period. Deaths were determined by word of mouth and a defaulter survey. Among all exited^e^ patients deaths accounted for 9.3% (139 of 1489 exits).

^a^1431 patients with new or established CVD were ever enrolled during the study period

^**b**^Including: amlodipine, atenolol, bisoprolol, enalapril, hydrochlorothiazide, valsartan; excluding: exclusively frusemide or spironolactone

^**c**^Among 51 asthma patients randomly selected for clinical audit

^d^Technically, the MSF guideline did not require cholesterol testing to be performed before calculating a CVD risk score, but qualitative data confirmed most clinicians waited for cholesterol results before calculating it

^e^Exited patients refers to those that were known to have died, were lost to follow up despite efforts to trace them or who had informed the team that they would no longer be attending the MSF service

#### Clinical indicators

Among 4044 adult patients meeting our inclusion criteria (i.e. diagnosed with hypertension and/or Type II diabetes (DMII) and enrolled during the study period), 2912 (72.0%) had hypertension and 2546 (63.0%) had DM II, while 1530 (37.8%) had a dual diagnosis. Within the programme’s first 6 months, mean systolic blood pressure decreased by 12.4 mmHg from 143.9 mmHg (95% CI 140.9 to 146.9) to 131.5 mmHg (95% CI 130.2 to 132.9) among hypertensive patients, while fasting glucose improved by 1.12 mmol/l, from 10.75 mmol/l (95% CI 10.04 to 11.47) to 9.63 mmol/l (95% CI 9.22 to 10.04), among type II diabetic patients. The probability of achieving treatment target in a visit was 63–75% by end of 2017, improving with programme maturation but with notable seasonable variation. From the patient perspective, the mean SBP in hypertensive patients decreased by 6.6 mmHg within the first 6 months, from mean 137.9 mmHg (95% CI 137.1 to 138.7) at entry/new diagnosis to 131.3 mmHg (95% CI 130.3 to 132.3) Similarly, there was a marked improvement in FBG level by 1.43 mmol/l from a mean of 10.40 mmol/l (95% CI 10.19 to 10.62) at entry/new diagnosis to 8.97 mmol/l (95% CI 8.67 to 9.26) by 6 months; most of this improvement occurred within the first 3 months. These results and those related to treatment interruption are elaborated on in our companion paper [38].

#### Quality indicators

Additional clinical outcome and process indicators are presented in Table 2. At each health education session patients were asked to categorise their exercise level as active, inactive, moderately active, and moderately inactive but exercise was not otherwise quantified. Activity levels did not seem to improve significantly. We could not determine whether smoking behaviour had changed since it was not quantified and patients’ self-reported smoking behaviour change was only recorded relative to their previous visit. Some activities were under-performed such as statin prescribing, CVD risk scoring and performance of annual urinary protein testing in diabetic patients. There appeared to be good levels of asthma control with only 2.6% of patients with asthma reportedly having an exacerbation within the preceding 6 months. However, rates of statin prescribing were low for patients with CVD (25%).

#### Perceived effectiveness

Interviewed staff and patients perceived the programme as effective. Patients reported feeling physically and psychologically better after attending the programme, linking this to having a regular supply of medications and some relief of their financial burden. A Jordanian patient noted: *“I feel relieved and comfortable since the first day I came here, I felt the difference in my disease.”*

### Implementation

Under this domain, we examined the fidelity of programme delivery, the challenges and facilitators to implementation, the subsequent adaptations made and the costs of programme delivery.

#### Fidelity of programme delivery

Indicators exploring fidelity of programme implementation are presented in Table 3 and were determined via routine cohort data analysis and clinical audit.

**Table 3:** Implementation indicator results

	Result or comment
**Process Indicators**	
% HTN patients with annual FBG performed	Not available (not calculated)
% DM patients^a^ with annual eye check performed	Annual^b^ fundoscopy documented OR referred for retinal screening = 50.8%
% of DM patients^a^ with micro-albuminuria or urinary protein tested	Annual^b^ Albumin creatinine ratio checked in 83.8%
% of DM patients^a^ on ACE inhibitor with creatinine checked	Annual^b^ creatinine check in 98.5%
% of active cohort with health education session at last clinical visit	66.9%^c^
Number of MHPSS group sessions monthly	Average 5.5 per month in 2016 and 2017
% of referred patients attending MHPSS individual counselling	Not available as number of internal MHPSS referrals was not captured
**Adaptations**	
Number/% of follow-up consultations performed by nurses	6% in 2017

Key: *ACE* angiotensin-converting enzyme, *FBG* fasting blood glucose, *HTN* hypertension, *MHPSS* mental health and psychosocial support

^**a**^Among 130 randomly selected diabetic patients’ charts analysed for the clinical audit.

^**b**^Annual referred to the 12 months preceding their most recent appointment.

^**c**^Among patients active in 2017 (*n* = 4011)

Health education was reportedly not delivered as intended. Clinical supervisors described the staff’s style as “*didactic*”, “*harsh*” and “*combative*”. Staff used a knowledge-based approach with patients, which involved *“telling them what to do”*, whereas a “solution-focused” approach and motivational interviewing techniques were preferred:

“*(Using) words like ‘you are not being honest’, ‘I don’t feel like you’re telling the truth’,’ if you only would’ … doesn’t work… This concept of patient-centred care, solution focused therapy, it’s what works.”* Clinical supervisor.

#### Challenges and facilitators

Here we present the challenges and facilitators related to patient access, implementation and maintenance that led to the specific adaptations detailed in the following section. Specific individual-level challenges around adherence to medications and healthy living advice are discussed later.

For patients, the profound impact that war and the refugee experience had on Syrian refugees’ lives proved to be the key challenge to delivering effective NCD care to these patients. Syrian patients’ psychological distress, social suffering and poverty had enormous implications for their ability to access and engage with the programme, as explored in detail in our linked paper [32]:

*“The hypertension goes high … when I get sad and remember my sons in Syria and they tell me what happens with them I keep crying and crying then my hypertension goes high or goes down… I take a hypertension pill to settle down whenever I read some news about them.”* Syrian patient.

The challenges reported by clinical staff also related to Syrians’ experience of war. Many staff clearly stated that they could not manage medical problems in isolation from the psychosocial issues patients faced. They felt ill-equipped to deal with Syrian patients’ war-related trauma and found it personally challenging. They highlighted the added complexities involved in treating Syrian versus Jordanian patients due to their perceived lower education and literacy levels and limited “hope” for the future. Care delivery was also complicated by the culture of private medical care and patients’ care seeking behaviour, with both nationalities tending to visit multiple concurrent providers and to prefer branded medication. MSF introduced an appointment system, contrary to common practice in Jordan, and patients’ initial failure to adhere to appointments proved frustrating for staff. Clinical and supervisory staff discussed the challenges inherent in providing chronic NCD care, such as long consultation times and dealing with the complexity of multi-morbid patients, especially those with renal failure. They noted that frail, elderly or housebound patients found physical access to both clinics sites difficult (via stairs). Finally, staff also described contextual and cultural challenges around healthy living education and behaviour change. These included diet and exercise norms (high fat, high salt diet and low habituation to exercise for health or leisure), the acceptance of smoking (especially in men), the obesogenic environment and most patients’ reliance on medications to provide solutions.

Staff perceived that facilitators to programme implementation included excellent patient-staff rapport, positive experiences of supervision, support and training, and good teamwork with colleagues. The MSF NCD guideline reportedly facilitated implementation and was largely acceptable and “useful”. Staff found it comprehensive and adaptable to the local context, serving as a tool to negotiate patient demands. However, clinical staff also highlighted the limited guidance on complex multi-morbid patients, while management staff requested additional programmatic guidance on defining a primary level NCD package (“what components are included…that is not clear”) and predicting referral needs. Jordanian doctors reportedly perceived the guideline as limiting their autonomy and offering “second-class” generic medication. Several called for a digital version facilitating access via smart phone.

#### Adaptations

Interviewed management and clinical staff described how the programme, designed around a high-income country primary care model, adapted dynamically to identified patient, programmatic and contextual challenges. The major adaptations are listed below:

- The MHPSS service was an essential addition to the programme. It was initiated in response to high rates of mental ill health among Syrian patients and limited adequate referral options. Starting with individual counselling sessions, it was later expanded and reoriented to provide ad hoc psycho-education sessions in waiting rooms, peer-support groups and a targeted group ‘living well’ programme combining health education and psychosocial support.
- By the end of 2017, only 0.5% (*n* = 24) of enrolled patients were formally diagnosed with a comorbid mental health condition and only 3.0% (*n* = 154) attended individual counselling sessions. Sixty-six group-counselling sessions were held in 2016, when recording began. (MHPSS service data did not capture numbers enrolled in group or waiting room sessions and were not linked to the general dataset). Most patients interviewed for this evaluation were unaware of the MHPSS services. Staff reported issues around social acceptability from both their own and patients’ perspectives and their reluctance to “label” patients as requiring MHPSS. Physical space, patient transport costs and limited patient engagement also proved barriers to patient engagement with MHPSS services. In response to the initial distrust and low rate of referrals from the programme doctors, the MHPSS undertook multidisciplinary staff training sessions and referral rights were extended to nurses.
- Depression screening was introduced and later paused as the numbers screening positive overwhelmed existing service capacity. At the time of the study, the team reported an ongoing lack of good quality referral options for patients requiring prescription of psychotropic medications or psychiatric input. Therefore, management staff planned to train one family medicine specialist and to expand MSF’s medication list to address this need.
- The humanitarian liaison officer’s social work role was introduced to address Syrians’ social and protection needs by linking them with other available services. It was reportedly underutilised as few referrals were made by the clinical team.
- Interviewed staff adapted health education messages to patients’ literacy and education levels, their limited financial means and their living environments. Staff also involved family members as informal treatment supporters.
- A home visit service was introduced in 2015 to improve access for elderly, housebound and frail patients. The team (a nurse, doctor and driver) initially served a 10 km radius from the clinics and both team and catchment area were later expanded.
- Management staff reported introducing clearer admission criteria relating to patient vulnerability.
- An appointment system with short message service (SMS) appointment reminders and an appointment tool were introduced to increase efficiency. Patients valued the reminders and the appointment system, which minimised the long waits and prevented the perceived favouritism they experienced in the MOH system. However, they also perceived it as rigid with services inaccessible outside of prescribed appointment times. Staff strongly encouraged patients to attend at their planned appointment day and time, achieving a 90% adherence rate by 2017.
- Task sharing to nurses of the care of “stable”, less complex patients achieving clinial control was introduced and stable patients’ appointment interval was increased from 1 to 3 months. Family medicine specialists were added to the team to support management of more complex patients. Task sharing had occurred in a very limited manner by the end of 2017 because of lack of clarity on clinical activity and patient flow, lack of clear eligibility criteria, reported resistance from patients and medical staff, national regulations limiting nurses’ roles. Increasing stable patients’ appointment interval to 3-monthly required dispensing of 3 months’ worth of medications. This necessitated the expansion of pharmacy team capacity.

#### Costs

The total annual financial cost of the NCD programme from the provider perspective increased annually in parallel with greater patient volume, greater service complexity and with the addition of specialist staff. It increased by 52% from INT$ 4,206,481 in 2015 to INT$ 6,400,611 in 2016 and by a further 5% to INT$ 6,739,438 in 2017. Per-patient-per-year (PPPY) cost increased 23% from INT$ 1424 (2015) to 1751 (2016), and by 9% to 1904 (2017), while cost per consultation increased from INT$ 209 to 253 (2015–2017). The major cost drivers were human resources (accounting for 38.9–42.6% of total annual costs) and medications (34.8–43.2%). The costs are reported in detail in a related paper [20].

### Maintenance

Under the *Maintenance* domain, we explored the challenges and facilitators related to programme maintenance at the individual and organisational level.

#### Individual level

We explored retention in care, medication burden, challenges and supports around psychosocial issues and adherence to medication and healthy living advice. Routine cohort data analysis showed that the majority of patients enrolled during the study period (*N* = 5045) were retained in care for over 6 months, with 85% attending a follow-up appointment six-months (+/− 30 days) after enrolment; while one-third of enrolled patients had exited (including 12.5% cumulative loss to follow up and 2.6% deaths) (Table 2).

Over half of adherence survey participants (*N* = 300; 74.4%) were prescribed four or more MSF-provided medications (Supplementary material S4B). The majority (60.4%) also took medications obtained from another source. Most patients (89%) had very high self-reported medication adherence scores. While the majority of individual interview participants (especially Syrians) declared themselves “very committed” to taking medications, several described stopping, taking intermittently or sharing medications with those in need. Staff and patients both emphasised the negative impact of mental distress on adherence to medications and healthy living advice:

*“As I was hearing the stories I thought…this man’s problem is not that he’s smoking too much. His problem is that he … experienced sexual violence, physical violence in prison in Syria… these two are linked.”* Clinical staff member.

Qualitative data confirmed that patients’ medication adherence and behaviour change was facilitated by support from family and MSF staff.

#### Organisational level

Here we explored the costs, challenges faced and possible modifications necessary to maintain the programme at organisational and contextual levels. With senior management, we discussed the lessons learned that could improve the programme or facilitate its scale-up, transfer or adaptation to other settings.

Our costing data supported our interviewees’ impression that this was an expensive programme. To support programme planning, we explored potential cost savings that could be achieved by varying the organisation of medical consultation workflow, which we presented in a related paper. The frequency of patient contact with the facility had the greatest influence on cost-savings; as more patients were categorised as “stable”, they were thus more suitable for less expensive nurse review and for longer review intervals [20].

Many of the challenges elicited were related to delivering chronic care to a conflict-affected population in a refugee setting with all the attendant psychosocial, physical and financial challenges. This proved the key challenge to implementing and maintaining effective NCD care in the Syrian refugee population.

MSF staff and stakeholders described the programme as being delivered within the framework of a complex and fragmented humanitarian system. Staff struggled to assist patients in navigating an often opaque, frustrating and unresponsive referral system.

“*The credibility of any service…depends on its ability to refer upwards…That is just as true for people with angina … (as it is) for mental health.”* Management staff member.

Referral pathways were limited by: cost (MSF and UNHCR covered limited essential conditions, procedures and providers); inconsistent availability (some referral services provided on short-term project bases); and bureaucracy (MSF was required to refer to MOH services via an intermediary).

In addition, the programme operated in a middle-income country of the Middle East with well-established health systems, regulations and policies, which tightly regulated humanitarian actors’ activities. Policies included the requirement that medications must be locally purchased (which increased costs); the lack of government focal point or set of regulations governing NGOs; significant bureaucratic delays; and strict regulation (for example around prescribing of psychotropic medications, nurse-prescribing and permission for Syrian clinicians to practice in Jordan).

In terms of facilitators of programme maintenance, management staff highlighted that the availability of highly qualified Jordanian professional staff facilitated implementation of this complex, multidisciplinary model of care but that this level of staffing would be unavailable in other settings where MSF works.

Qualitative data highlighted the importance placed by MSF staff on providing a good quality service that fulfilled MSF’s humanitarian remit. There was a perceived tension between their desire to continually improve the programme and the need to consider long-term planning and a potential future handover. While the MOH was considered by some management staff as the likely handover partner, they emphasised its limited capacity and the gulf between current MSF and MOH models of NCD care.

The internal debate within MSF around the appropriateness of a humanitarian NGO engaging in chronic NCD care and their relative inexperience in doing so also posed its own unique challenge to the maintenance of the programme, as described here:

*“An NCD Programme is a relatively recent departure for MSF and it is getting very close to the dividing line between humanitarian and development aid. (There is a) general sense among the humanitarian community that NCDs are an epidemic and need to be dealt with, but I am not sure we have …(a clear) view of how this should be managed...”* Management staff member.

Several MSF management staff noted the particular challenge involved in adapting MSF’s more familiar approach, characterised as providing relatively short-term solutions to health care gaps in populations in crisis, to the setting of chronic disease care. Several also questioned the sustainability and/or the potential to hand over the complex Irbid care model and mentioned the Jordanian MOH and other NGOs as potential hand over partners. However, senior MSF staff highlighted the rationale for maintaining the specific vertical programme in Irbid. It served as an opportunity for MSF to “learn by doing” and to understand the essential components of NCD care. To continue operating in the Jordanian context, a middle-income country with established systems, regulations and policies, required a different type of engagement and negotiation with authorities compared to other contexts where MSF has traditionally worked, which may have fewer resources and weaker systems.

Several staff members suggested that MSF could engage more closely with pre-existing health systems in designing future NCD interventions, and could build on their HIV service model, by maximising task sharing and decentralisation of care to community level.

## Discussion

Our mixed methods evaluation guided by the RE-AIM framework has helped to characterise the implementation strategies, challenges and adaptations made to a complex, multidisciplinary intervention providing primary level NCD care in a humanitarian setting.

### Programme coverage, acceptability and access to chronic care in Jordan

The MSF Irbid NCD Programme provided free-of-charge care to a limited patient cohort, covering approximately one quarter of the target adult Syrian population and a number of Jordanians. Enrolled patients’ NCD risk factors and disease prevalence reflected regional norms [19, 30, 39, 40]. The programme was largely acceptable to patients, staff and stakeholders, although patients were frustrated by the siloed approach to care and limited access to referral services.

One key finding, consistent with the literature, was the lack of access to affordable NCD care both for non-MSF patients and for MSF patients seeking care for conditions not covered by MSF [19, 29–31]. Syrian refugees’ access to NCD care was likely diminished following Jordanian government policy to significantly increase their MOH co-payments to “foreigner” levels in 2018, which was later reversed in 2019 [31]. McNatt et al. reported that, following the policy change, NCD patients increasingly sought care from the NGO rather than MOH sector, attending multiple providers to create comprehensive NCD care for themselves. Patients in their study found this process financially and emotionally burdensome. MSF could also note their finding that the burden of indirect costs of clinic attendance (transport, lost work time) potentially outweighed the benefits of free NGO-provided care [31].

### Delivering chronic care in a humanitarian setting

Many of the challenges in programme implementation encountered by MSF were related to a humanitarian organisation delivering chronic disease care to a conflict-affected population. The impact of Syrian patients’ experience of war, loss and social suffering on their engagement with NCD care was a key finding [32]. The lack of accessible and consistent specialist care referral pathways for NCD complications in this context has been described in the literature [29, 41]. MSF's temporary solution via other international NGOs was dependent on short-term project-based funding. For future NCD programme design, we recommend attempting to secure essential referral pathways (e.g., ophthalmology, cardiology, nephrology) that are acceptable, accessible and affordable for patients, and linked directly with MOH services, where possible. We acknowledge that this may be extremely challenging, especially in low-income countries with constrained health systems, and would require agreement on financing, clear referral criteria and continuity of information. Strengthening NCD care within the MOH system in humanitarian settings also requires greater focus and financing of NCDs by major donors. Other challenges identified were related to the specific Jordanian context, a middle-income country with well-established health systems, regulations and policies, which tightly regulated humanitarian actors’ activities compared to other settings with weaker systems.

### Key programme adaptations

This MSF programme repeatedly adapted to patient and programmatic needs. Key adaptations included the addition of a specific, culturally-relevant MPHSS service, the introduction of the HLO social work role and the development of specific referral criteria for MHPSS, social work and external services [32]. There appeared to be scope to further improve both patient education (by taking a more solution-focused approach, utilising patients’ own strengths, skills and intrinsic motivation) and medication adherence support [42]. Further work is needed to develop adherence measurement and support tools in this population but joint decision making with patients and involving treatment supporters may prove valuable, as found in other contexts [43].

MSF also adapted recall and data collection tools to chronic care delivery by introducing specific appointment times, appointment reminders, individual patient files and a patient-level electronic database. The latter allowed for cohort analysis, as previously demonstrated by the UN Relief and Works Agency for Palestinian refugees (UNRWA) [44–46]. Key lessons included the need for a fit-for-purpose and actionable information system and the need to establish informative indicators without overburdening staff with data collection.

### Effectiveness of the programme

The programme appeared to achieve good intermediate clinical outcomes for hypertension and diabetes. These findings reflect those reported by MSF and UNRWA in similar humanitarian settings [21, 44, 47]. However, it should be noted that we know little about the prevalence or outcomes of major complications of these illnesses, such as heart failure, ischaemic heart disease and peripheral vascular disease. This is partly because these conditions are difficult to measure at primary care level, requiring equipment and trained personnel, but also because of the limited affordable specialist care available to MSF patients for diagnosis of these conditions in Jordan [29, 48]. The apparently good asthma control outcomes relied on patient self-report and may have reflected poor recording of this variable. The low rate of statin coverage is an important finding. Since this is a proven, effective strategy to reduce mortality, we suggest that further staff training on CVD secondary prevention, further audits (ideally as part of a quality improvement strategy), and the introduction of fixed dose combination CVD secondary prevention drugs may boost statin prescribing [48–50]. In terms of hard outcomes, such as mortality, 2.6% of the cohort was known to have died during the study period. This is not surprising given this was an elderly population with multi-morbidity and limited access to specialist care. It may be underreported, since most deaths took place in hospitals or in the community and, in many cases, cause of death was not known. Further study of death rates and cause of death is warranted, necessitating longer follow up periods.

### Maintaining the programme

We identified a number of key challenges to maintaining the programme and areas for further improvement. Principal among these was cost. MSF management staff perceived the programme to be costly but, to our knowledge, there are no available published data to directly compare the programme’s costs with similar services, either in the Middle East region or in other humanitarian settings. This programme was more costly than MSF-reported *incremental* PPPY costs of adding NCD care to existing services in Mweso, Democratic Republic of Congo [INT$222 (2015)] and in Eswatini [INT$441 (2016)] [7, 33]. Limited data on NCD care from countries affected by the Syrian crisis have focused on the costs of secondary or tertiary level care [51–53]. High costs were at least partly responsible for MSF limiting the service’s coverage and scope. However, some adaptations introduced by MSF, triaging the cohort patients by disease complexity and control, introducing task sharing to nurses and spacing review appointments for stable patients could result in cost savings, as discussed elsewhere [20]. It was possible to employ family medicine specialists to manage the more complex patients in Irbid because of the availability of highly qualified Jordanian staff, but such staff would likely be unavailable in many humanitarian settings with more constrained health systems. It is therefore essential to provide programmatic and clinical written guidance appropriate to different contexts, which could potentially be supported by technology, such as telemedicine and/or mHealth decision support tools, as trialled by other actors in Lebanon [54].

Humanitarian actors’ modus operandi is to rapidly identify needs and bring healthcare to vulnerable or marginalised populations, then withdraw or hand over activities, as the context dictates. This approach is not consistent with the continuous care required for chronic conditions and may explain interviewed participants’ apparent discomfort with the lack of a “handover strategy” and “vertical” nature of the programme. Senior staff emphasised the role the Irbid programme played as one of MSF’s pilot NCD-specific programmes, serving both to anchor the organisation in Jordan and as a training programme. While the programme served MSF well as a learning ground it was unlikely to be scalable in Jordan or reproducible in other humanitarian settings, mainly due to cost and the required numbers and skill mix of staff.

MSF and other humanitarian actors recognise that integration of NCD care within existing health systems, ideally at primary care level, may be the optimal approach [2]. Integration may provide an opportunity for health system strengthening, particularly in contexts where resource-poor health systems have previously focused on episodic emergency or infectious disease care and have limited capacity to provide chronic disease care [1, 55, 56]. Designing future NCD services may require a comprehensive analysis of the pre-existing health system’s readiness to manage NCDs, particularly at primary care level, and its resilience in the face of crises.

### Lessons learned and potential solutions

The lessons learned and adaptations made as the programme evolved may be relevant to MSF, the MOH and other humanitarian actors and may be transferable to other settings. A number of approaches are interlinked and could potentially achieve several things: increased patient-centeredness, increased cost-efficiency for patients and provider, and increased coverage. These goals could be achieved by reducing facility-based contact through decentralisation, enhancing community-level care and supporting patient self-management. These approaches could involve further task sharing to nurses or other non-physician health worker cadres, such as community workers or volunteers. Several aspects of the care pathway could be shifted to the community level, including prevention and sensitisation activities, diagnosis, treatment monitoring and adherence support. Patient centeredness (taking a holistic, responsive approach and actively collaborating with patients and families) could involve either providing “one stop shop” comprehensive primary care at a single facility visit or bringing care to the patient via outreach workers or home care teams [34, 57, 58]. Adherence and self-management could be supported via mobile phone or wearable technology or through peer support groups led by community workers or peers [56]. Clearly, the specific design and the successful implementation of these strategies would be context-dependent and would rely on local acceptance by patients, staff and the medical fraternity as well as political and regulatory support.

Several actors in Jordan have introduced community-based healthy living interventions or peer support groups for people with diabetes on project or pilot bases [59–62]. Some reported positively impacting intermediate clinical outcomes, such as weight and blood glucose levels. However, cost effectiveness, sustainability, acceptability or user experiences were not formally examined. The recently published HOPE4 trial also demonstrated the benefits of a community-based package of care for hypertension in a non-humanitarian setting [63].

## Strengths and limitations

To the best of our knowledge this is the first study to comprehensively describe a mixed-methods evaluation of an NCD service in a humanitarian setting guided by the RE-AIM framework. It builds on our previous use of the framework in the Democratic Republic of Congo [6, 7]. We made comprehensive use of RE-AIM, addressing each of the domains and including more extensive explanatory qualitative and costing analyses than are often employed in the RE-AIM literature [15]. We could comment in only a limited way on *adoption and participation* as they have been traditionally used, since this intervention took place at a single site rather than involving multiple sites/providers. We included process indicators relevant to Quality of Care in several domains. Alternatively these could be grouped under the “Implementation-fidelity” subdomain.

The challenges of conducting research in humanitarian contexts, the need to improve evaluation of humanitarian programmes in general and the lack of evidence describing the effectiveness of NCD care models in humanitarian settings have previously been noted [3, 10–13, 64]. We demonstrated that implementation research can be conducted while placing limited burden on staff and patients. We also highlighted the challenges in retrospectively evaluating humanitarian programmes, which tend to be highly responsive to changing contexts, and in analysing routinely collected data. For example, it was not feasible for us to include a comparator group or use a quasi-experimental design, such as interrupted time series, given the dynamic and unique nature of the programme. Indicators designed for this evaluation have contributed to the ongoing development by humanitarian organisations of a set of shared NCD indicators. A number of our indicators could not be measured due to failure to collect or limited usability of data and we emphasise the need to co-develop indicators with implementers, especially when using routine programmatic data.

We note there is a need to replicate this model to distinguish what is essential to this site rather than essential across settings. We also note our limited understanding of the situation of people with NCDs who did not reach care, for instance, those who were undiagnosed, who attended irregularly, or who could not physically access services. Similarly, we did not interview patients currently attending MOH or other NGO services, although our findings about alternative NCD services reflect those of other authors [31]. We note, finally, that social desirability bias may have influenced results of the qualitative data and of the self-report medication adherence survey, which was mainly administered by the data collectors rather than by patients as intended.

## Future research and evaluation

As discussed, there is a need to design and perform implementation research around the streamlined high-quality NCD programme models described above in humanitarian settings, particularly facilitating access for mobile or dispersed populations. Designing and evaluating novel ways to improve access to diagnosis and management of NCD complications at primary care level is also essential, which could include use of telemedicine, mobile technology or artificial intelligence-supported diagnosis or clinical decision tools [48, 54, 64]. We recommend that future research should focus on elucidating programme impact, where possible, using methods such as causal inference frameworks and prospective interrupted time series analyses. Longer study durations would facilitate examination of hard outcomes, such as cardiac events and deaths. Further exploration of access and quality of care issues, utilising patient quality of life and satisfaction outcomes and disaggregating by sex, would also be useful. In addition, patient-level costing studies, examining direct and indirect patient costs, and cost-effectiveness studies are lacking.

## Conclusion

RE-AIM has proven a valuable tool to guide the evaluation of a complex intervention in a protracted humanitarian crisis setting. Most elements of the MSF programme were perceived as acceptable to patients, staff and stakeholders, whereas adaptations were required to improve the acceptability of the MHPSS services. It was accessible and affordable for the programme’s cohort of enrolled patients, while achieving good intermediate clinical outcomes. However, the programme had limited coverage and the current model was both costly and complex and therefore challenging for other actors to emulate or to translate to other, more financially constrained settings. We propose that simplification of the care model, reduction of costs and use of technology could improve effectiveness and efficiency without reducing acceptability and may improve transferability to other settings.

## Key recommendations

1. **Patient-centred**. Adopt a contextualised, patient-centred approach where possible. For example, deliver care at community level, support patients and families to self-manage and provide holistic, “one-stop-shop” care at facility visits. Elicit and respond to patient priorities. In this case they were: consistent, affordable medication and respectful and caring staff.
2. **Complex, yet efficient care.** There is a broad range of patient complexity involved in NCD care, from asymptomatic hypertensive patients to frail, elderly patients with complex disease involving polypharmacy and multi-morbidity. It is important to acknowledge this complexity and the holistic approach needed when drafting guidelines and designing services. Consultations are time consuming and patients may require frequent review. Where appropriate, a context-adapted, algorithm-driven approach may facilitate task sharing to nurses of the stable, less complex patients. Introducing fixed dose combination pills, for example, may reduce pill burden and ease adherence, while simplifying prescribing and workload in relevant settings.
3. **Continuum of care.** NCDs require a continuum of care involving primary prevention, diagnosis and treatment, prevention and management of complications, psychosocial support, rehabilitation and palliation. A multi-disciplinary team would ideally deliver this package of care, where available.
4. **Mental health and psychosocial support** should be included as an integral part of primary level NCD services in humanitarian settings. This may be integrated or provided by partner organisations. Provide a tiered approach to MHPSS according to need: 1. Basic support available to all, 2. Psychosocial or peer support groups for specific patient groups (such as teenagers with diabetes), and 3. Individualised counselling and medical intervention.
5. **Adapted healthy living advice.** Adapt advice to patients’ constrained circumstances and use proven techniques such as solution-focused counselling and motivational interviewing.
6. **Access to referral services.** A predictable proportion of patients will require referral for screening, diagnosis or treatment of NCD-related complications. However, it may be difficult to secure essential referral pathways (e.g. ophthalmology, cardiology, nephrology) that are acceptable, accessible and affordable for patients. Therefore, it is essential to maximise the quality of primary NCD care to prevent, identify and effectively manage complications.
7. **Low cost to patients yet cost-efficient for providers**. The ideal way to ensure access is to provide free-of-charge care to patients, where possible. The model of NCD care presented here was relatively costly from the provider perspective, especially in terms of HR and drugs. We have shown that savings could be made by reducing the frequency of facility-based contact and by introducing context-adapted procurement practices.
8. **Health system strengthening.** Integrate with host health systems where possible and engage in health system strengthening appropriate to the local context, in order to ensure sustainability and facilitate movement of patients from private to state health systems. This may require a comprehensive analysis of the pre-existing health system readiness to manage NCDs, particularly at primary care level, and its resilience in the face of crisis, before embarking on an NCD intervention.
9. **Monitoring and evaluation adapted to chronic care.** Implement more broadly the structures, reporting mechanisms and indicators developed within the MSF Irbid programme to reflect the needs of a chronic disease programme.
10. **Research.** Engage patients and stakeholders in the design and evaluation of new models of NCD care in humanitarian settings. These may involve simplification, greater use of task sharing, decentralisation of care to the community level, and use of technology for patient and provider support.

## Supplementary Information

**Additional file 1. **Summary of MSF Irbid NCD programme REAIM evaluation indicators and methods. **A)** Table showing: main indicators relevant to each RE-AIM domain and the methods and data sources used to determine them, and **B)** Figure showing: schematic representation of methodologies and indicators.

**Additional file 2.** Intervention description for MSF NCD programme in Irbid, north Jordan. Detailed description of the MSF NCD programme, including enrolment criteria, elements of clinical management, patient circuit and follow up pattern.

**Additional file 3.** Programme Timeline MSF NCD programme in Irbid, north Jordan. Timeline showing key contextual and programmatic change during the study period December 2014 to December 2017.

**Additional file 4. **Medication Adherence Survey Additional Material. Tables showing: **A)** Medication adherence data collection and analysis, **B)** Demographics of 300 adult patients of the Irbid NCD programme who responded to a medication adherence survey in September 2017 and **C)** Proportions of answers to individual questions of MARS-5 questionnaire; 1D. Frequency of sum scores for MARS-5 from 300 survey patients from Irbid NCD Clinic.

**Additional file 5. **Qualitative Study Additional Material. **A)** Detailed description of the qualitative study methods for MSF Irbid NCD programme evaluation, **B)** Participant list for patient, staff and stakeholder semi-structured individual interviews and **C)** Topic guides for focus group discussion with patients and individual interviews with patients, health care providers and staff and stakeholders.

**Additional file 6. **Cohort Study Demographics, Cardiovascular Risk Factors at Enrolment and NCD Diagnoses at Last Visit. Tables showing: **A)** Demographics by country of origin of 5029 Syrian and Jordanian patients enrolled in Irbid NCD Programme 2014–2017, **B)** Cardiovascular Risk Factors at Enrolment for the cohort 2015–2017 and **C)** Per patient diagnoses at last visit for all patients enrolled in the Irbid NCD Programme 2015–2017 by age and gender.

## Data Availability

The datasets used and/or analysed during the current study are available from the corresponding author on reasonable request, with the permission of Médecins sans Frontières (ocaresearch@london.msf.org) and under a data sharing agreement.

## Abbreviations

BP: Blood pressure
BMQ: Beliefs About Medicines Questionnaire
COPD: Chronic obstructive pulmonary disease
CVD: Cardiovascular disease
DM: Diabetes mellitus
FBG: Fasting blood glucose
FGD: Focus group discussion
HbA1c: Glycosylated haemoglobin
HLO: Humanitarian liaison officer
HTN: Hypertension
INT$: International dollars
JHAS: Jordan Health Aid Society
LMIC: Low- and middle-income countries
MARS-5: Medication Adherence Report Scale-5 item
MENA: Middle East and North Africa
MOH: Ministry of Health
MSF: Médecins sans Frontières
MHPSS: Mental health & psychosocial support
NCD: Non-communicable disease
NGO: Non-governmental organisation
RE-AIM: Reach, effectiveness, adoption, implementation and maintenance
SMS: Short message service
UNHCR: United Nations High Commissioner for Refugees
UNRWA: UN Relief and Works Agency for Palestinian Refugees
WHO: World Health Organization
