# Supplementary material for: MSF experiences of providing multidisciplinary primary level NCD care for Syrian refugees and the host population in Jordan: an implementation study guided by the RE-AIM framework
Source: BMC Health Serv Res. 2021 Apr 26;21:381. doi: 10.1186/s12913-021-06333-3 (PMC8074194; doi:10.1186/s12913-021-06333-3)
Supplement: Supplementary file 1 — Additional file 1. Summary of MSF Irbid NCD programme REAIM evaluation indicators and methods. A) Table showing: main indicators relevant to each RE-AIM domain and the methods and data sources used to determine them, and B) Figure showing: schematic representation of methodologies and indicators. [file 12913_2021_6333_MOESM1_ESM.docx]

**Supplementary Material S1**

**Title: Summary of MSF Irbid NCD programme REAIM evaluation indicators and methods**

**Description: A)** Table showing: main indicators relevant to each RE-AIM domain and the methods and data sources used to determine them, and **B)** Figure showing: schematic representation of methodologies and indicators

**A) Full list of indicators and data method/source based on the RE-AIM domains**

| Objective / Domain (Questions) | Sub-Domain | Indicator | Methods  (a methodology may feature under several headings) |
| --- | --- | --- | --- |
| Reach  Does the programme reach its target population? | Coverage | Number people among the target population eligible for programme  Number served by the programme  Representativeness of those reached  Prevalence of NCD and MH comorbidity; eligibility for MHPSS services; numbers referred/receiving care; representativeness of those reached | Existing MSF cross-sectional survey ^  Routine cohort data  Qualitative data |
| “Effectiveness”/ Quality of Care  What are the trends in clinical outcomes and quality indicators of the programme?  What are the perceived benefits/unintended consequences from a patient and provider perspective? | Clinical Outcomes | No./% HTN patients with most recent BP <= 140/90, 6 & 12 months post enrolment and trend from baseline*  No./% of DM patients with most recent BP <= 140/90, 6 & 12 months post enrolment and trend from baseline*  No./% of patients with diabetes with last HbA1c < 8.0 % 6 & 12 months post enrolment and trend from baseline*  No./% with cholesterol reduction >= 0.5 mmol/L from baseline at 6 and 12 months post enrolment  No./% of patients with asthma / COPD free from exacerbations/ admissions in previous 6 months  No./% of patients who report decreased/quitting smoking  No./% of patients who report increased levels of exercise from baseline  Trend in referrals to another facility for acute complications/specialist care | Routine cohort data  Qualitative data |
|  | Quality Indicators | Proportion of referrals to other services that are appropriate per guideline  No./% active^¶^ CVD patients prescribed a statin  No./% active^¶^ CVD patients prescribed aspirin  No./% active^¶^ CVD patients prescribed at least one anti-hypertensive  No./% COPD/ asthma patients with inhaler technique check documented  No./% appropriate clinical action taken based on clinical/ laboratory findings  Trend in defaulters* as a proportion of active cohort during reporting period  Description of cohort deaths (patient characteristics) | Clinical audit  Routine cohort data |
|  | Perceived Effectiveness | Patients’ and providers’ perspectives on effectiveness of programme components (clinical review, medications, HE, HLO, MHPSS, HV) | Qualitative data |
| Adoption/ acceptance  Is the MSF model of NCD care accessible and acceptable to patients, providers, organisation and community?  Is the MSF NCD guideline acceptable to staff? | Accessibility/ acceptability | Duration and frequency of NCD service and components  Staff (e.g. ratio of staff per patient)  Structures and tools  Treatment continuity/rupture  Staff and patient perceptions of availability and accessibility / barriers to access of service components (clinical review, HE, HLO, MHPSS, HV)  Staff perspectives on acceptability/usability of NCD guideline  Key stakeholder views on acceptability/accessibility MSF NCD programme  Self-reported medication adherence levels and medication beliefs | Routine cohort data  Qualitative data  Self-report medication adherence questionnaire |
|  | Adoption/  participation | Description of intervention location, cadres of staff and qualifications; inclusion/exclusion criteria of staff/settings delivering service  Sources & perception of information/support  Experience of receiving and providing NCD care, use of clinical guideline  How participation influenced patient/staff well-being and/or work practices | Routine cohort data  Qualitative data |
| Implementation  To what extent was the intervention delivered as intended?  What are the facilitators and barriers to implementing the programme from a patient, provider and programmatic perspective?  What are the essential components and adaptations necessary to delivering an NCD service in this setting?  What are the start-up and incremental costs of delivering such a service? | Fidelity of programme delivery  (Process Indicators;  indicators in bold also reflect quality of medical care) | Extent to which clinical guideline delivered as intended:  No./% of eligible patients with HTN with annual FBS performed during the reporting period  No./% of eligible patients with diabetes that have had an annual foot check/ eye check performed during the reporting period  No./% of DM patients that have micro-albuminuria or urinary protein testing during the reporting period  No./% of DM patients on ACE inhibitor (ACEi) with creatinine testing during the reporting period  No./% asthmatics and COPD with control review (spirometry or clinical) during the reporting period  No./% of active cohort attending a health education session at last clinical visit within reporting period  No. of MHPSS group sessions taking place monthly during reporting period  No./% of referred patients attending MHPSS individual counselling sessions  No./% of times when clinical action taken based on clinical or laboratory findings according to guideline | Clinical audit  Routine cohort data |
|  | Adaptations | NCD care adaptations to the local setting (e.g. cultural adaptations; dietary and exercise, smoking advice)  Programme adaptations related to humanitarian setting e.g. response to patients’ psychosocial needs | Qualitative data |
|  | Cost | Staff time  Capital and recurrent implementation costs ^#^ | Qualitative data  Medicine/supply/ staff costs^#^  Staff time estimates^#^ |
| Maintenance  What are the challenges and facilitators for patients to remain in the programme?  What are the costs involved in maintaining the programme?  What are the programmatic challenges and adaptations made to maintain the programme? | Individual Level | No./% patients active^¶^ 6 months post enrolment *  No. medications and daily pill count at last consultation  Self-reported medication adherence rates and medication beliefs  Qualitative measure of individual-level maintenance:   - Key challenges in maintaining medical treatment (including medication concordance) - Key challenges in altering lifestyle (diet, exercise, smoking) - Key mental health/ psychosocial challenges - Types of support available and strengths and challenges of the support (health education, MHPSS, HLO, family and community support) | Routine cohort data  Clinical Audit  Qualitative data  Medicine/supply/staff costs^#^  Staff time estimates  Self-report medication adherence questionnaire |
|  | Organisational Level | Measures of cost of maintenance^#^  Institutionalisation of the programme/modifications made for maintenance  Alignment with organisational mission |  |

**Key:** ACEi=angiotensin converting enzyme inhibitor; BP=blood pressure; COPD=chronic obstructive pulmonary disease; CVD=cardiovascular disease; FBS=fasting blood sugar; HLO=humanitarian liaison officer; HV= home visit; MHPSS=mental health and psychosocial support; NCD=non-communicable disease. ^ The methods and results pertaining to these indicators are reported in Rehr et al (25). * The methods and results pertaining to these indicators are reported in our linked paper (30). **^#^** The methods and results pertaining to these indicators are reported in our linked paper (28). **^¶^** Active patients referred to those that have continued to attend the service and have not exited (i.e. died, departed the area or defaulted (i.e. have not attended for more than 90 days since their last planned appointment)

**B) MSF Irbid NCD Evaluation Schematic representation of methodologies and indicators (reproduced and adapted from De Silva *et al* ^30^)**
