# Supplementary material for: MSF experiences of providing multidisciplinary primary level NCD care for Syrian refugees and the host population in Jordan: an implementation study guided by the RE-AIM framework
Source: BMC Health Serv Res. 2021 Apr 26;21:381. doi: 10.1186/s12913-021-06333-3 (PMC8074194; doi:10.1186/s12913-021-06333-3)
Supplement: Supplementary file 2 — Additional file 2. Intervention description for MSF NCD programme in Irbid, north Jordan. Detailed description of the MSF NCD programme, including enrolment criteria, elements of clinical management, patient circuit and follow up pattern. [file 12913_2021_6333_MOESM2_ESM.docx]

**Supplementary Material S2**

**Title: Intervention description for MSF NCD programme in Irbid, north Jordan:**

**Description:** Detailed description of the MSF NCD programme, including enrolment criteria, elements of clinical management, patient circuit and follow up pattern.

The multidisciplinary team initially included non-specialist doctors, nurses, health educators, pharmacy and reception staff. The team provided appointment-based medical consultation, incorporating health education (HE) and behaviour change counselling, and were supported by a local management team and a coordination team in Amman. The service evolved to also incorporate individual and group-based mental health and psychosocial support (MHPSS), social work, physiotherapy and a home visit team for house-bound patients. Further staff roles were added including counsellors and a specialist family medicine practitioner at each clinic site as well as a physiotherapist, a humanitarian liaison officer (HLO), who performed a social work role, linking patients with protection and other humanitarian services, and the pharmacy and home visit teams were expanded (Programme Timeline S3).

Most patients (>90%) presented with established, self-reported diagnoses on enrolment; new diagnoses were made based on the MSF NCD guideline (35). The MSF guideline was adapted from international guidance and WHO PEN and Primary Care International guidance, both specifically developed for resource-poor and humanitarian settings (68–70). At first visits, doctors recorded a complete past medical, medication and family medical history and performed a clinical examination. In addition, lifestyle CVD risk factors (smoking status, alcohol intake, exercise levels) were recorded; the global cardiovascular risk score was calculated using WHO CVD risk prediction chart; acute complications were identified and treated; long-term medications were prescribed for symptom management and secondary prevention of complications; patients were referred for laboratory testing as appropriate; and a follow-up interval was determined (71).

Patients were initially reviewed on a monthly basis and followed a defined patient circuit involving a registrar/clerk, triage nurse, doctor, health educator and the pharmacy team. Clinics ran six days per week from 8 am to 2 pm, while the home visit service operated on six days within a ten-mile radius of the clinics. Ton increase programme efficiency, policy for stable patients changed in 2016. Their clinical review appointments were task-shared to nurses and the review interval was increased to three months. However, medications were still dispensed monthly, so they were required to attend monthly to collect them. Stable patients were defined as those achieving the programme’s clinical targets i.e. blood pressure < 140/90 mmHG; FBG < 180 mg/dL or HbA1c < 8%; and clinically controlled asthma, COPD or angina, as relevant to the patient.

*Complications screening and referral.* Hypertensive and CVD patients had annual fasting capillary blood glucose (FBG) performed to screen for diabetes. Clinic staff measured diabetic patient’s blood pressure and capillary blood glucose at each visit and screened for micro-and macrovascular complications with annual foot checks (examination and monofilament testing) and referral to an external laboratory for annual microalbuminuria, serum creatinine and cholesterol testing.

Referrals pathways were complex, changed over time and were made to multiple other actors. Referrals for specialist care or care for non-target NCDs (e.g. musculoskeletal disease or cancer) were not funded by MSF. Emergency cases were stabilised (e.g. acute asthma exacerbation, diabetic ketoacidosis, acute coronary syndrome) and were then referred to the Jordanian public health emergency services by ambulance. Non-urgent referrals (most frequently ophthalmology, cardiology and nephrology) were made to public, private or other humanitarian providers. MSF’s referral patterns varied greatly over time as the availability of services, e.g. NGO-provided cardiac catheterisation, depended on short donor funding cycles. UNHCR oversaw the funding for registered refugees and covered MOH primary care and limited secondary, tertiary and emergency care according to strict eligibility criteria. Appointments were noted in an appointment book; clinical data were collected in paper-based files and entered weekly into a patient-level, macro-based Excel spread sheet by a data entry operator.
