# Supplementary material for: MSF experiences of providing multidisciplinary primary level NCD care for Syrian refugees and the host population in Jordan: an implementation study guided by the RE-AIM framework
Source: BMC Health Serv Res. 2021 Apr 26;21:381. doi: 10.1186/s12913-021-06333-3 (PMC8074194; doi:10.1186/s12913-021-06333-3)
Supplement: Supplementary file 4 — Additional file 4. Medication Adherence Survey Additional Material. Tables showing: A) Medication adherence data collection and analysis, B) Demographics of 300 adult patients of the Irbid NCD programme who responded to a medication adherence survey in September 2017 and C) Proportions of answers to individual questions of MARS-5 questionnaire; 1D. Frequency of sum scores for MARS-5 from 300 survey patients from Irbid NCD Clinic. [file 12913_2021_6333_MOESM4_ESM.docx]

**Supplementary Material S4**

**Medication Adherence Survey Additional Material**

**1A. Methods:** *Data collection and analysis*

Our 17-item self-report medication adherence survey included questions about demographic information (gender, nationality, age group, education level, household size, number of NCD medications, and number and sources of other medications) and the utilised pre-existing medication adherence and beliefs about medicines tools. The Medication Adherence Survey-5 (MARS-5) is a five-item self-report measure of medication adherence. The Beliefs about Medicine Questionnaire – Specific (BMQ-S) is a 10-item self-report measure exploring people’s beliefs about their current medications.

Respondents were asked to rate the frequency they engage in each of the adherence-related behaviours on a five-point scale, where 5 = ‘never’, 4 = ‘rarely’, 3 = ‘sometimes’, 2 = ‘often’ and 1 = ‘always’. Scores for each item are summed to give a total score; higher scores indicate higher levels of reported adherence. The MARS-5 may be used to distinguish between intentional and unintentional adherence, which may guide intervention strategies to improve adherence. The BMQ-S consists of 10 statements about medications scored using a 5-point Likert scale (from 1 = strongly disagree to 5 = strongly agree). Five questions relate to the perceived *necessity* of medications and five relate to patients’ *concerns* about taking medication.

Frequencies, mean, median and standard deviations were calculated for the individual and sum scores of the MARS-5. For the BMQ, the total per patient scores for “necessity” questions (possible scores 5 to 25) and “concerns” questions (possible scores 6 to 30) were calculated. The individual MARS5 scores were transformed to a 0 to 1 scale to allow for logistic regression. For each variable, the MARS total and individual question scores and the BMQ necessity and concerns scores were described using univariate analysis. P-values for heterogeneity were applied. Multivariate logistic regression analysis tested the effect on adherence of medication beliefs (necessity–concerns difference scores AND necessity scores AND concerns scores), demographic variables (age, gender, nationality, marital status, educational experience, household size) or clinical factors (number of diagnoses, number of prescribed medicines, number of medication sources).

**1B. Demographics of 300 adult patients of the Irbid NCD programme who responded to a medication adherence survey in September 2017.**

| Variable | Category | n | % |
| --- | --- | --- | --- |
| Age (years) | <50 | 73 | 24.3 |
|  | 50-59 | 100 | 33.3 |
|  | 60-69 | 86 | 28.7 |
|  | 70+ | 41 | 13.7 |
| Gender | Male | 99 | 33.0 |
|  | Female | 136 | 45.3 |
|  | Not answered | 65 | 21.7 |
| Nationality | Jordanian | 87 | 29.0 |
|  | Syrian | 212 | 70.7 |
| Marital status | Married | 240 | 80.0 |
|  | Other | 12 |  |
|  | Widow(er) | 48 | 16.0 |
| Education Level | None | 42 | 14.0 |
|  | Primary | 191 | 63.7 |
|  | Secondary + | 90 | 28.0 |
| Diagnoses | Diabetes | 212 | 70.7 |
|  | Asthma | 11 | 3.7 |
|  | Lung disease | 2 | 0.7 |
|  | Thyroid | 28 | 9.3 |
|  | Hypertension | 230 | 76.7 |
|  | CVD | 109 | 36.3 |
|  | Other | 51 | 17.0 |
| No NCD Diagnoses | 1 | 84.0 | 28.0 |
|  | 2 | 118.0 | 39.3 |
|  | 3+ | 98.0 | 32.7 |
| MSF medications | 1-3 | 76.0 | 25.3 |
|  | 4-6 | 152.0 | 50.7 |
|  | 7-15 | 72.0 | 24.0 |
| No other sources of meds | 0 | 121.0 | 40.3 |
|  | 1 | 162.0 | 54.0 |
|  | 2+ | 17.0 | 5.7 |

**1C. Proportions of answers to individual questions of MARS-5 questionnaire**

Per cent of respondents

**1D. Frequency of sum scores for MARS-5 from 300 survey patients from Irbid NCD Clinic**

Number of patients

Mars-5 Sum Score
