# Supplementary material for: MSF experiences of providing multidisciplinary primary level NCD care for Syrian refugees and the host population in Jordan: an implementation study guided by the RE-AIM framework
Source: BMC Health Serv Res. 2021 Apr 26;21:381. doi: 10.1186/s12913-021-06333-3 (PMC8074194; doi:10.1186/s12913-021-06333-3)
Supplement: Supplementary file 5 — Additional file 5. Qualitative Study Additional Material. A) Detailed description of the qualitative study methods for MSF Irbid NCD programme evaluation, B) Participant list for patient, staff and stakeholder semi-structured individual interviews and C) Topic guides for focus group discussion with patients and individual interviews with patients, health care providers and staff and stakeholders. [file 12913_2021_6333_MOESM5_ESM.docx]

**Supplementary Material S5**

**Title: Qualitative Study Additional Material**

**Description: A)** Detailed description of the qualitative study methods for MSF Irbid NCD programme evaluation, **B)** Participant list for patient, staff and stakeholder semi-structured individual interviews and **C)** Topic guides for focus group discussion with patients and individual interviews with patients, health care providers and staff and stakeholders.

**A) Detailed description of the qualitative study methods for MSF Irbid NCD programme evaluation**

The qualitative study involved two same-sex focus group discussions (FGDs) with eight Syrian adult patients each and forty individual semi-structured interviews (SSI): sixteen with adult Syrian and Jordanian patients, eighteen with MSF staff, and seven with key stakeholders.

Patients scheduled for medical review during a 2-week period were stratified by NCD diagnosis and then randomly selected by study staff to be invited to participate in an interview or FGD, held at times convenient to patients. Syrian and Jordanian patients were eligible for interviews, while programme staff recommended that Syrians alone were included in FGDs to avoid participants feeling inhibited by the presence of Jordanians. Additional patients were purposively selected from clinic waiting rooms to ensure both sexes, both main nationalities and those accessing each clinic location and specialised service element (MHPSS, HLO, Home Visit) were represented. MSF staff were purposively selected to represent a range of clinical, support and managerial staff, past and present. More medical staff than other staff cadres were selected to evaluate the acceptability and implementation of the MSF NCD guideline. Key stakeholders were selected to represent different levels of the MOH, other NGOs involved in delivering NCD care in north Jordan and a representative of the Syrian community.

Qualitative data were collected in August 2017. All invited participants agreed to participate and signed an informed consent form. We conducted individual patient interviews until data saturation was achieved, which resulted in a relatively small sample since we were interested in broader, over-arching themes rather than in fine-grained themes. The number of staff and stakeholder interviews were based on practical time limitations but theoretical saturation was felt to have been reached. A topic guide included introductory questions about the patient’s NCD or the participant’s role in relation to NCD care and questions relating to each domain of the RE-AIM framework. We focussed on specific components of NCD care (e.g. service provision, clinical consultation, medication prescription and adherence, health education, MHPSS and/or support from the HLO, home visit service). The English-language FGD and SSI topic guides are included as Supplementary material S3. All FGDs, patient interviews and three staff interviews were conducted in Arabic by two trained research assistants (HT, male, current HLO; SE, female, former HLO) at MSF clinics or in one patient’s home. The remaining interviews were conducted in English by EA (female, public health researcher at LSHTM) at MSF premises, stakeholders’ offices, or via Skype for former MSF staff. In each case, participant privacy was assured. Interviews were audio-recorded, translated and transcribed by a study team member with quality checks performed by a second team member. Patient interviews included nine male and seven female patients, of whom ten were Syrian and six were Jordanian. The majority (n=13) had two or more NCD diagnoses, three had attended MHPSS services, two attended the HLO and one was a home visit patient (Supplementary material S3).

Data were coded in NVivo11© and analysed by EA and a co-analyst using template analysis whereby a coding template was developed, based on an initial subset of data, then applied to further data and refined iteratively (72,73). This allowed for an integrated approach employing both deductive and inductive coding. Deductive coding was framed around the *a priori* themes based on RE-AIM (13,14). Data were analysed by participant subset, i.e. patient, staff or stakeholder, and were checked with reflexive practice to mitigate against the insertion of preconceived assumptions. Themes were then related back to the research question and to existing literature. Negative cases or exceptions were examined to explore what set them apart. Both analysts reviewed the final template to enhance inter-rater reliability and analytic credibility.

**B) Participant list for patient, staff & stakeholder semi-structured individual interviews**

| **PATIENT** | | | |
| --- | --- | --- | --- |
| **Code** | **Diagnosis** | **Origin** | **Gender** |
| **PT01** | DM, HTN | Jordanian | Male |
| PT02 | DM, HTN | Jordanian | Female |
| PT03 | DM | Syrian | Female |
| PT04 | CVD asthma | Jordanian | Male |
| PT05 | DM, CVD, MH | Syrian | Male |
| PT06 | DM, HTN, | Syrian | Male |
| PT07 | HTN | Syrian | Male |
| PT08 | DM, HTN | Syrian | Male |
| PT09 | DM | Jordanian | Female |
| PT10 | MD, MH, HLO | Syrian | Female |
| PT11 | DM, asthma, MH | Syrian | Female |
| PT12 | DM, CVD | Syrian | Male |
| PT13 | DM (wife of patient) | Jordanian | Female |
| PT14 | DM, HTN | Jordanian | Male |
| PT15 | DM, HTN, CVD, HLO | Syrian | Female |
| PT16 | HTN, CVD, HV | Syrian | Male |
| **STAFF** | | | |
| **Code** | **Role** | | **Gender** |
| ST01 | Psychosocial counsellor | | Female |
| ST02 | Psychosocial counsellor | | Female |
| ST02 | Registrar (reception staff) | | Female |
| ST04 | Doctor | | Female |
| ST05 | Doctor | | Male |
| ST06 | Health educator | | Female |
| ST07 | Nurse | | Male |
| ST08 | Pharmacist | | Female |
| ST09 | Doctor | | Female |
| ST11 | Medical Coordinator | | Female |
| ST12 | Project Coordinator | | Female |
| ST13 | Mental Health Activities Manager | | Female |
| ST14 | Medical Activities Manager | | Male |
| ST15 | Medical Activities Manager | | Male |
| ST16 | Project Coordinator | | Male |
| ST17 | Medical Coordinator | | Male |
| ST18 | Pharmacist | | Male |
| **STAKEHOLDERS** | | | |
| **Code** | **Role** | | **Gender** |
| KS01 | NGO | | Male |
| KS02 | MOH District | | Male |
| KS03 | MOH District | | Female |
| KS04 | MOH Clinic | | Male |
| KS05 | MOH Central | | Male |
| KS06 | Syrian Community Member | | Male |
| KS07 | NGO | | Female |

Key: CVD=cardiovascular disease; DM=diabetes mellitus; HLO=humanitarian liaison officer; HTN=hypertension; HV=home visit; MH=mental health and psychosocial support service; MOH=Ministry of Health; NGO=non-governmental organisation.

**C) Qualitative Study Focus Group Discussion and Individual Interview Topic Guides**

**C1. Topic guide – Focus group discussions with NCD patients**

| Key area | Themes | Question |
| --- | --- | --- |
| Introduction | Study aim and agencies involved | Why invited to participate? Consent & any questions? |
| Participant Background | Getting to know each other + building rapport | Could you tell us a bit about yourself? *Prompt: e.g. profession, what area live in, when you were first diagnosed with [NCD condition]?* |
| Reach | Access  Barriers to accessing care for NCDs  Ways of reducing barriers | What do you know about your NCD condition(s)? *Prompt – e.g. causes, types, who gets it, treatment*  Could you tell me about how you came to learn about your NCD condition(s)? ? *Prompt – e.g. from friends/family, from the radio (or other media), when diagnosed at hospital.*  What were you told about NCD condition(s) when you were enrolled in the MSF clinic? *Prompt – probe understanding of NCD condition(s) such as causes, risks and its management (medication and diet).*  What do you think might prevent people from attending this service for their NCD condition(s)? *Prompt: lack of knowledge, lack of services, costs, time, quality of services, stigma etc.*  How could access to healthcare for NCD condition(s) be improved? |
| Adoption and implementation | Information  Support | How did you feel when you were enrolled into this clinic? *Prompt: counselling/support experience. Prompt: subsequent days/weeks experience*  Who did you talk to about your experience at the clinic? *Prompt: E.g. family members, friends.*  What were you told about managing your NCD condition(s) after you were enrolled in clinic (by the NCD staff)? *Prompt: medicine types and usage, managing medicines, diet changes, risks and symptoms, frequency of check-ups etc.*  What sources of support did you receive in managing your NCD condition? *Prompt: emotional support from family/friends, information support from health workers, MHPSS from health workers.*  What made it easier for you to access care – initially and continuing care?  What made it difficult for you to access care – initially and continuing care?  What made it easier for you to self-manage your NCD condition at home?  What made it difficult for you to self-manage your NCD condition at home?  How acceptable do you find the NCD service / treatment. *Prompt: e.g. logistically, socially, culturally etc., differences with previous experience of treatment/ service?*  What has been your experience of the psychosocial services offered by the programme – group sessions/individual counselling*?* |
| Maintenance | Challenges  Supportive factors  To support adoption and implementation | What have been the main challenges in maintaining your medical treatment for your NCD condition? *Prompt: time, costs, information, drug supply, pill burden, stigma/shame etc.*  What have been the main challenges in altering your diet? *Prompt: information, costs, support*  What have been the main challenges in increasing your levels of exercise? *Prompt: information, suitable facilities or locations, physical condition, support, costs*  What have been the main challenges in reducing or quitting smoking? *Prompt: information, support, costs, desire*  What could have made accessing care easier for you? *Prompt: e.g. information given – content and way it was delivered; costs; type and quality of care and support; focus on role of the NCD programme/services.*  What could have made achieving lifestyle changes easier for you? *Prompt: e.g. information given – content and way it was delivered; costs; type and quality of care and support; focus on role of the NCD programme/services;*  What support is available to help you to continue to attend the clinic and self-manage your condition?  What additional supports regarding your NCD condition would you like to have? |
| Effectiveness | Unintended consequences  Benefits | What have been the negative consequences of taking NCD treatment / attending the service? *Prompt: physical, psych, costs, time.*  What have been the benefits of receiving NCD treatment / attending the service? *Prompt: e.g. physical, psychological, social, economic.*  What have been the benefits or negative consequences of attending group sessions/ individual counselling? |
| Thanks and  close | Anything else to add  Questions/Thanks, feedback info | Anything else to add on topic that we haven’t discussed today? Any questions for me? Feedback again on how the discussion will be used and fed back. |

**C2. Topic guide – Semi-structured interviews with NCD patients**

| **Key area** | **Themes** | **Question** |
| --- | --- | --- |
| Introduction | Study aim and agencies involved  Why invited to participate  Consent & any questions? |  |
| Participant Background | Getting to know each other + building rapport | Could you tell us a bit about yourself? *Prompt: e.g. profession, what area live in, when you were first diagnosed with NCD condition?* |
| Reach | Knowledge in community  Access to testing for [NCD condition]  Barriers to testing | What do you know about your condition? *Prompt – e.g. causes, types, who gets it, treatment*  What has been your experience in accessing healthcare and medications for your condition? *Prompt – in Syria, in Jordan, other NGOs or clinics, why choose to come to MSF clinic, does experience differ?*  Do you think a lot of people have your NCD condition in your community?  What do you think prevents people from accessing healthcare/medications for NCD conditions?  What do you think would make it easier for people to access healthcare / medications for NCD conditions?  If MSF were not providing this service what would you do to manage your condition? |
| Adoption and implementation | Information and other support provided  Adjusting to condition | What type of information provided to you about your condition and its treatment when you were enrolled in the MSF clinic? *Prompt: causes, who gets it, chronic nature, medicine types and usage, managing medicines, diet changes, risks and symptoms, frequency of check-ups etc.*  What sources of support did you receive? *Prompt: emotional support from family/friends, information support from health workers, psychosocial support from health workers*  What other support would you have liked to receive?  Do you find it easy to come in to the clinic from the beginning? *Prompt: facilitators or barriers e.g. logistically, socially, culturally*  How has having your NCD condition changed your daily life/routine? *Prompt: e.g. difficulties in changing your daily routine, in Syria or in Jordan?*  What is your experience of the MHPSS part of the programme (group sessions or individual counselling)? |
| Maintenance | Barriers/challenges to adhering to appointments and prescribed medicine/ lifestyle change. | Do you come in regularly for all your appointments? Do you find it easy or difficult to do so? Why? (e.g. travel, time, stigma)  What is your experience when you come to the clinic?  Do you experience any difficulties when you are visiting the clinic for follow up?  What could be done to make it easier for you to come to the clinic?  Do you take your medicines as often as you are prescribed? Why? *(Prompt: don't think it's important, unsure how to take them, can’t read the instructions, too many pills, share with family/ friends, supply rupture)* Do you find it easy or difficult to do so? Why? (*Prompt* *difficult to remember)*  Do you feel any pressure not to take your medicines (*Prompt: stigma from family or community, cost, medication sharing)*  What could be done to make it easier for you to take your medications?  Do you find it easy to maintain the recommended diet, exercise levels, smoking cessation for your condition?  What has helped you to make lifestyle changes? Prompt: health education, medical staff, family or community support?  What challenges do you face in adapting your diet, exercise levels and smoking habits?  Do you thing MHPSS is important?  What challenges do you face in taking part in or attending MHPSS support (group sessions, or individual counselling)? |
| Effectiveness | How coming to the clinic has affected patient's condition | What have been the negative consequences of taking treatment for the condition/ attending the service? *Prompt: physical, psych, costs, time.*  What have been the benefits of receiving treatment for your condition / attending the service? *Prompt: e.g. physical, psychological, social, economic.*  *What have been the positive and negative consequences for you in attending the MHPSS sessions (group or individual counselling)? (Prompt: feel supported, feel better, assists with managing NCD condition, upsetting, difficult)* |
| Thanks and  close | Anything else to add  Questions/Thanks, feedback info |  |

**C3. Topic guide – Semi-structured interviews with NCD health care providers and staff**

| **Key area** | **Themes** | **Question** |
| --- | --- | --- |
| Introduction | Study aim and agencies involved  Why invited to participate  Consent & any questions? |  |
| Participant Background | Getting to know each other + building rapport | Could you tell us a bit about yourself? Prompt: e.g. professional, involvement in the NCD service at Irbid (and previously if relevant)? |
| Reach | Access  Barriers to NCD care provision  Ways of reducing barriers | What are the key challenges for patients to access healthcare (medications, regular clinical review, investigations, interventions) for their NCD condition(s) e.g. knowledge, costs, time, availability or quality of care [expand], stigma etc.  How could access to healthcare for NCD condition be improved? Prompt: improve knowledge (e.g. outreach, radio, health workers etc.), improve availability of services, quality of services etc. |
| Adoption and implementation | Information and support | What types of information are provided to patients when they are enrolled in/ attend the NCD service?  What sources of support are offered to patients when they are enrolled in/ attend the NCD service)?  How acceptable do you think the MSF NCD programme, including treatment, is for patients? Prompt: e.g. quality, responsiveness, socially, culturally etc.  What is your experience with implementing the new MSF NCD guideline?  What sources of support and information were available to you to facilitate implementing the guideline? |
| Maintenance | Challenges  Supportive factors  To support adoption and implementation | What do you think are the main challenges facing NCD patients here in terms of managing their condition? Prompt: medicines/testing/attendance - time, costs, information, drug supply etc.; lifestyle changes – knowledge, social/cultural pressures etc.  What could be done to make it easier for NCD patients to access care? Prompt: e.g. information given – content and way it was delivered; costs; type and quality of care and support; [note: focus on role of the [NCD condition] programme/services].  What do you think are the main challenges facing staff here in terms of delivering the NCD care programme in Irbid? Prompt: time, training, clinical support/supervision, guidelines or tools  What could be done to make it easier for staff to deliver this NCD care programme? Prompt: knowledge, time, training, clinical support/supervision, guidelines or tools  What are the benefits of using the MSF NCD guideline?  What are the challenges around using the MSF NCD guideline?  What could be done to facilitate implementation of the guideline? |
| Effectiveness | Unintended consequences  Benefits | What are the benefits of the NCD care programme in Irbid? Prompt: more efficient, less complications, for patients, for staff, for system, for community etc.  What are negative consequences of the NCD care programme in Irbid? Prompt: time, complexity, costs etc. for patients, for staff, for system, for community  What particular aspects of the programme have helped or hindered NCD care? Prompt: clinical aspects, task shifting, introduction of HLO, MHPSS, HV, structures, tools, systems  What particular aspects of the guideline have helped or hindered NCD care? Prompt: supports decision making, ease of use, contradictory, not acceptable to patients, different to usual practice in Jordan. |
| Thanks and  close | Anything else to add  Questions  Thanks, feedback info | Anything else to add on topic that we haven’t discussed today?  Any questions for me?  Feedback again on how the discussion will be used and fed back. |

**C4. Topic guide – Semi-structured interviews with key stakeholders**

| **Key area** | **Themes** | **Question** |
| --- | --- | --- |
| Introduction | Study aim and agencies involved  Why invited to participate  Consent & any questions? |  |
| Participant Background | Getting to know each other + building rapport | Could you tell us a bit about yourself? Prompt: e.g. professional, involvement in the NCD service at Irbid (and previously if relevant)? |
| Reach | Access  Barriers to NCD care provision  Ways of reducing barriers | What are the key challenges for patients to access healthcare (medications, regular clinical review, investigations, interventions) for their NCD condition(s) e.g. knowledge, costs, time, availability or quality of care [expand], stigma etc.  How could access to healthcare for NCD condition be improved? Prompt: improve knowledge (e.g. outreach, radio, health workers etc.), improve availability of services, quality of services etc. |
| Adoption and implementation | Information and support | How acceptable do you think the MSF NCD programme, including treatment, is for patients? Prompt: e.g. quality, responsiveness, socially, culturally etc. |
| Maintenance | Challenges  Supportive factors  To support adoption and implementation | What do you think are the main challenges facing NCD patients in this area in terms of managing their condition? Prompt: medicines/testing/attendance - time, costs, information, drug supply etc.; lifestyle changes – knowledge, social/cultural pressures etc.  What could be done to make it easier for NCD patients to access care? Prompt: e.g. information given – content and way it was delivered; costs; type and quality of care and support; [note: focus on role of the [NCD condition] programme/services].  What do you think are the main challenges facing staff here in terms of delivering the NCD care programme in Irbid? Prompt: time, training, clinical support/supervision, guidelines or tools  What could be done to make it easier for staff to deliver NCD care programme? Prompt: knowledge, time, training, clinical support/supervision, guidelines or tools  What are the benefits of using the MSF NCD guideline? [ask if appropriate]  What are the challenges around using the MSF NCD guideline? [ask if appropriate]  What could be done to facilitate implementation of the guideline? [ask if appropriate] |
| Effectiveness | Unintended consequences  Benefits | What are the benefits of the NCD care programme in Irbid? Prompt: more efficient, less complications, for patients, for staff, for system, for community etc.  What are negative consequences of the NCD care programme in Irbid? Prompt: time, complexity, costs etc. for patients, for staff, for system, for community  What particular aspects of the programme have helped or hindered NCD care? Prompt: clinical aspects, task shifting, introduction of HLO, MHPSS, HV, structures, tools, systems  What particular aspects of the guideline have helped or hindered NCD care? Prompt: supports decision making, ease of use, contradictory, not acceptable to patients, different to usual practice in Jordan. |
| Thanks and  close | Anything else to add  Questions  Thanks, feedback info | Anything else to add on topic that we haven’t discussed today?  Any questions for me?  Feedback again on how the discussion will be used and fed back. |
