# Supplementary material for: MSF experiences of providing multidisciplinary primary level NCD care for Syrian refugees and the host population in Jordan: an implementation study guided by the RE-AIM framework
Source: BMC Health Serv Res. 2021 Apr 26;21:381. doi: 10.1186/s12913-021-06333-3 (PMC8074194; doi:10.1186/s12913-021-06333-3)
Supplement: Supplementary file 6 — Additional file 6. Cohort Study Demographics, Cardiovascular Risk Factors at Enrolment and NCD Diagnoses at Last Visit. Tables showing: A) Demographics by country of origin of 5029 Syrian and Jordanian patients enrolled in Irbid NCD Programme 2014–2017, B) Cardiovascular Risk Factors at Enrolment for the cohort 2015–2017 and C) Per patient diagnoses at last visit for all patients enrolled in the Irbid NCD Programme 2015–2017 by age and gender. [file 12913_2021_6333_MOESM6_ESM.docx]

**6. Supplementary Material S6**

**Title: Cohort Study Demographics, Cardiovascular Risk Factors at Enrolment and NCD Diagnoses at Last Visit**

**Description:** Tables showing: **A)** Demographics by country of origin of 5029 Syrian and Jordanian patients enrolled in Irbid NCD Programme 2014- 2017, **B)** Cardiovascular Risk Factors at Enrolment for the cohort 2015-2017 and **C)** Per patient diagnoses at last visit for all patients enrolled in the Irbid NCD Programme 2015-2017 by age and gender.

**6A. Demographics by country of origin of 5029 Syrian and Jordanian patients enrolled in Irbid NCD Programme 2014- 2017**

| Variable | Category | Total | % | Syrian | % | Jordanian | % |
| --- | --- | --- | --- | --- | --- | --- | --- |
| *Country of Origin* |  | 5029 | 100 | 3664 | 72.9 | 1365 | 27.1 |
| *Gender* | Male | 2021 | 40.2 | 1429 | 39 | 592 | 43.4 |
|  | Female | 3008 | 59.8 | 2235 | 61 | 773 | 56.6 |
|  | Age <5 | 18 | 0.4 | 14 | 0.4 | 4 | 0.3 |
|  | Age 5-15 | 150 | 2.9 | 119 | 3.2 | 31 | 2.3 |
|  | Age 15-40 | 498 | 9.9 | 384 | 10.5 | 114 | 8.4 |
|  | Age 40-65 | 3035 | 60.3 | 2163 | 59 | 872 | 63.9 |
|  | Age >65 | 1328 | 26.4 | 984 | 26.9 | 344 | 25.2 |
| *Education level* | None | 724 | 14.4 | 613 | 16.7 | 111 | 8.1 |
|  | Not asked/answered | 1874 | 37.3 | 1472 | 40.2 | 402 | 29.5 |
|  | Primary | 1423 | 28.3 | 1123 | 30.6 | 300 | 22.0 |
|  | Secondary or higher | 1008 | 20 | 456 | 12.4 | 552 | 40.4 |
| *Household size* | 1-3 | 889 | 17.7 | 480 | 13.1 | 409 | 30.0 |
|  | 4-6 | 1743 | 34.7 | 1143 | 31.2 | 600 | 44.0 |
|  | 7+ | 2019 | 40.2 | 1738 | 47.5 | 281 | 20.6 |
|  | Not asked/answered | 378 | 7.5 | 303 | 8.3 | 75 | 5.5 |
| *Impaired mobility* | Yes | 498 | 9.9 | 358 | 9.8 | 140 | 10.3 |
|  | No | 4381 | 87.1 | 3207 | 87.5 | 1174 | 86.0 |
|  | Not asked/answered | 150 | 3.0 | 99 | 2.7 | 51 | 3.7 |

**6B: Cardiovascular Risk Factors at Enrolment for the cohort 2015-2017**

|  | Total (N=5045) | % | Male (N=2023) | % | Female (N=3022) | % |
| --- | --- | --- | --- | --- | --- | --- |
| Current smoker | 1144 | 22.7 | 825 | 40.8 | 319 | 10.6 |
| Inactivity (moderate/total) | 1879 | 37.2 | 560 | 27.7 | 1319 | 43.6 |
| Obesity (BMI>30)* | 2555 | 62.6 | 799 | 47.2 | 1756 | 73.5 |
| Current alcohol intake | 15 | 0.3 | 12 | 0.6 | 3 | 0.1 |

*Based on first available BMI measurement; N=4082 (1692 men and 2390 women).

**6C: Per patient diagnoses at last visit for all patients enrolled in the Irbid NCD Programme 2015-2017 by age and gender**

|  | Total | | | | | Age Category | | | | | | Gender | | | | | |
| --- | --- | --- | --- | --- | --- | --- | --- | --- | --- | --- | --- | --- | --- | --- | --- | --- | --- |
|  |  | | | | |  | | | | | |  | | | | | |
| NCD | **n=**  **5045** | **%** | **05-15 n= 151** | **%** | **15-40 n= 501** | | **%** | **40-65 n= 3041** | **%** | **>65 n= 1334** | **%** | | **Male n= 2023** | **%** | **Female n= 3022** | | **%** |
| Hypertension | 3047 | 60.4 | 3 | 2.0 | 303 | | 60.5 | 1796 | 59.1 | 945 | 70.8 | | 1135 | 56.1 | 1912 | 63.3 | |
| CVD total ^(1)^ | 1306 | 25.9 | 0 | 0.0 | 73 | | 14.6 | 700 | 23.0 | 533 | 40.0 | | 762 | 37.7 | 544 | 18.0 | |
| DM I ^(2)^ | 155 | 3.1 | 56 | 37.1 | 89 | | 17.8 | 10 | 0.3 | 0 | 0.0 | | 76 | 3.8 | 79 | 2.6 | |
| DM II | 2680 | 53.1 | 2 | 1.3 | 308 | | 61.5 | 1659 | 54.6 | 711 | 53.3 | | 1095 | 54.1 | 1585 | 52.4 | |
| Hypothyroid | 383 | 7.6 | 13 | 8.6 | 123 | | 24.6 | 207 | 6.8 | 40 | 3.0 | | 37 | 1.8 | 346 | 11.4 | |
| Asthma | 352 | 7.0 | 77 | 51.0 | 117 | | 23.4 | 124 | 4.1 | 34 | 2.5 | | 142 | 7.0 | 210 | 6.9 | |
| COPD ^(3)^ | 71 | 1.4 | 0 | 0.0 | 5 | | 1.0 | 35 | 1.2 | 31 | 2.3 | | 57 | 2.8 | 14 | 0.5 | |
| MSK ^(4)^ | 296 | 5.9 | 0 | 0.0 | 30 | | 6.0 | 169 | 5.6 | 97 | 7.3 | | 90 | 4.4 | 206 | 6.8 | |
| Neurological | 78 | 1.5 | 0 | 0.0 | 14 | | 2.8 | 40 | 1.3 | 24 | 1.8 | | 27 | 1.3 | 51 | 1.7 | |
| Obesity | 2555 | 50.6 | 13 | 8.6 | 449 | | 89.6 | 1606 | 52.8 | 487 | 36.5 | | 799 | 39.5 | 1756 | 58.1 | |
| DM II + HT ^(5)^ | 706 | 14.0 |  | 0.0 | 169 | | 33.7 | 437 | 14.4 | 100 | 2.2 | | 338 | 16.7 | 368 | 12.2 | |
| DM II + HT + CVD | 702 | 13.9 | 0 | 0.0 | 25 | | 5.0 | 374 | 12.3 | 303 | 6.6 | | 369 | 18.2 | 333 | 11.0 | |
| HT + CVD | 449 | 8.9 | 0 | 0.0 | 10 | | 2.0 | 241 | 7.9 | 198 | 4.3 | | 232 | 11.5 | 217 | 7.2 | |
